# Shifting networks and mixing metals: Changing metal trade routes to Scandinavia correlate with Neolithic and Bronze Age transformations

**DOI:** 10.1371/journal.pone.0252376

**Published:** 2021-06-16

**Authors:** Heide W. Nørgaard, Ernst Pernicka, Helle Vandkilde

**Affiliations:** 1 Department for Archaeology, Moesgaard Museum, Aarhus-Højbjerg, Denmark; 2 Department of Archaeology and Heritage Studies, School of Culture and Society, Aarhus University, Aarhus-Højbjerg, Denmark; 3 Curt-Engelhorn-Center for Archaeometry, Mannheim, Germany; University of Padova: Universita degli Studi di Padova, ITALY

## Abstract

Based on 550 metal analyses, this study sheds decisive light on how the Nordic Bronze Age was founded on metal imports from shifting ore sources associated with altered trade routes. On-and-off presence of copper characterised the Neolithic. At 2100–2000 BC, a continuous rise in the flow of metals to southern Scandinavia begins. First to arrive via the central German Únětician hubs was high-impurity metal from the Austrian Inn Valley and Slovakia; this was complemented by high-tin British metal, enabling early local production of tin bronzes. Increased metal use locally fuelled the leadership competitions visible in the metal-led material culture. The Únětice downfall c.1600 BC resulted for a short period in a raw materials shortage, visible in the reuse of existing stocks, but stimulated direct Nordic access to the Carpathian basin. This new access expedited innovations in metalwork with reliance on chalcopyrite from Slovakia, as well as opening new sources in the eastern Alps, along an eastern route that also conveyed Baltic amber as far as the Aegean. British metal plays a central role during this period. Finally, from c.1500 BC, when British copper imports ceased, the predominance of novel northern Italian copper coincides with the full establishment of the NBA and highlights a western route, connecting the NBA with the southern German Tumulus culture and the first transalpine amber traffic.

## 1. Introduction

This study tracks changes in metal trade networks over 2,500 years in southern Scandinavia, from the advent of the Neolithic c. 3800 BC until the apex of the Nordic Bronze Age (NBA) prior to 1300 BC. Southern Scandinavia’s rich Bronze Age societies were dependent on copper imported from exogenous sources throughout the 2100–500 BC period, a fact clearly established by recent lead isotope results [1–3]. Additionally, it was shown that the existing local copper deposits have not been exploited before the Middle Ages [3]. Much less clear, however, is to what extent the principal ore sources in use were coupled to specific trade routes, whether shifts in these trade routes occurred over time, and whether societal changes played a role. A first step towards answering these questions was that recent scientific advances have identified the metal-bearing regions in Europe that helped to gear the NBA [1,3–8] towards such accomplishments as elite burial mounds by the thousand, the horse-pulled sun-chariot from Trundholm, or the twin helmets from Viksø. The present article discusses the implications of 543 metal analyses covering the time span 3800–1300 BC, with a focus on 311 new and unpublished analyses mostly from the later end of that time span, 1600–1300 BC (Table 1). The large number of trace element and isotope analyses now available has brought within our reach a far greater precision in determining the metal’s provenance–an advantage the present study utilises.

**Table 1:** The generally accepted chronological phasing rests on a combination of object typologies, object assemblages in hoards and burials, and ^14^C ranges.

PERIOD	approx. ^14^C RANGE *	CULTURE & SOCIETY	METALS
**Earlier Neolithic**	3800–2850 BC	Funnel-beaker culture with copper objects and copper metallurgy c. 3800–3500 BC	faint rise
**Younger Neolithic**	2850–2300 BC	Corded Ware in-migrations and impact	bust
**Late Neolithic I** (LN I)	2350–2100 BC	West European Bell Beaker impact	faint rise
**Late Neolithic II** (LN II)	2100/2000-1700 BC	First full metal-use and intensified social competition. Impact from EBA Classic Únĕtice and EBA Britain ‘Wessex I’	clear rise
**NBA IA**	1700–1600 BC	Metallurgical progress comprising full tin-bronze. Toward 1600 BC: demise or crisis alongside Únĕtice final collapse	continued rise
**NBA IB**	1600–1500 BC	NBA breakthrough: individualised hierarchy on rise: first big mounds. Refined metalwork. Impact from MBA Carpathian Basin	boom
**NBA II**	1500–1300 BC	NBA social consolidation and cultural *floruit*. Impact from Southern German Tumulus Culture.	boom

*The ^14^C ranges are based on a combination of radiocarbon and dendrochronological determinations [9–12].

The first 1,700 years were characterised by on-and-off presence of small amounts of metal. With improved data, the variation range of metal supplies to a region itself devoid of copper ore deposits can now be charted. Cross-European contacts co-stimulated early metallurgical and societal development between 2100 and 1300 BC in the north. At the peak, 1500–1300 BC, plentiful sophisticated objects of bronze and gold were invested in lavish rituals and personal aggrandisements. Measured by numbers and intensity of metal depositions, the Nordic region was one of the richest in Bronze Age Europe.

In southern Scandinavia, metalwork first appeared in the Neolithic Funnel-Beaker period, mostly in the shape of flat axes [13,14]. Evidence was recently reported for early Nordic metallurgical activity, at some point between 3800–3500 BC [15]. Following this, however, there is a break or even an absence of metal finds for the remainder of the Earlier Neolithic and the entire Younger Neolithic [16]. The number of metal finds rises in the early Late Neolithic period (LN I) in association with appropriations of the Bell-Beaker culture: on a limited scale, southern Scandinavia was active among incipient metal-using societies from c. 2350 BC (LN I) [17]. It was however not until the following Late Neolithic period (LN II), c. 2100–2000 BC, that this region became a fully metal-using society, participating in the formation of a globalised Bronze Age world [17]. From this point of no return, the amount of metal imported to southern Scandinavia began to grow in LN II and NBA IA. By 1600–1500 BC in NBA IB, huge amounts of metal were reaching southern Scandinavia, coinciding with the breakthrough of the NBA as an exceedingly rich cultural zone, despite its situation at Europe’s northern fringe [1,17–22]. During the bloom period of NBA II (1500–1300 BC), imports of metals increased continually, along with the consolidation of a new social order [5,23,24].

The much-improved data now available allows us to comprehensively review the metal flows to Scandinavia that stimulated the emergence of the full-grown NBA between 1600 BC and 1300 BC. Three recent developments in the data are crucial. Firstly, prior to 2016 the early formative period (pre-1500 BC) was underrepresented in isotope-based scientific studies, which prioritised the later NBA [3,6]. To model changes in the early transition of metal procurement and usage in Scandinavia, the project presented here aimed to obtain and evaluate many new analyses, especially from the relatively neglected first seven hundred years of continuous metallurgical achievements in southern Scandinavia. The novelty of the project rests on a tight triple combination of fine-meshed archaeological typology and understanding of technological traditions with trace element and lead isotope analyses of the metal. This article presents part two of its large dataset as an overall evaluation; part one was published in 2019 [1]. Secondly, plenty of geochemical analyses of major copper deposits in central and south-east Europe have now been published [25–39], in addition to key datasets of metal analyses of artefacts [8,40,41]. Thirdly, we build on the outline provided by the initial results of the project [1] to broaden and advance insight and knowledge in light of the much-enlarged dataset and recently recognised mobility patterns [42,43]. Moreover, the mechanisms at work in this decisive era of metallurgical activity in southern Scandinavia have now been modelled from a Nordic perspective at different scales on a spectrum from the local scale to the Bronze Age global scale [18,19,44].

Central to our inquiry into the commencement of the Bronze Age in southern Scandinavia is the question whether some form of correlation existed between ore source (copper type), trading route, and the constitution of NBA society. The three-way connection we postulate is investigated through key case studies. We assume that shifts in major copper mining fields may have had implications for long-distance metal-driven trade. Likewise, a major alteration in the European trading network would have had consequences for the NBA society, because regular metal supplies were necessary to sustain the political economy. The question then emerges whether the sequence of distinct sociocultural transformations at 2100–2000 BC (the turn of LN I to LN II), 1600 BC (the turn of NBA IA to NBA IB), and 1500 BC (the turn to NBA II) correlated with changes in the provenance and class of copper arriving in southern Scandinavia. The demise of the prominent Únětice culture at around 1700 BC and its cessation altogether c. 1600 BC [17,45,46] may serve as an example, in that it may well have expedited direct Scandinavian access to copper in Slovakia and the eastern Alps.

## 2. Material

The present study builds on data published in 2019 [1,2] comprising approximately 50% of all metal finds of the LN II and NBA IA (2100–1700 BC). For the present paper, an additional 311 artefacts were typologically examined in detail [4,47], analysed for major and trace element concentrations and lead isotope ratios, and critically evaluated and compared with similarly analysed ores from Europe (S1 and S2 Tables). Thus, the overall dataset has been extended by the inclusion of data from NBA IB and Early NBA II, 1600–1450 BC, a point widely accepted as the breakthrough and consolidation of the NBA. Also added are eleven new samples from the Earlier Neolithic and Younger Neolithic, one sample from LN I, seventeen samples from LN II, and two more from NBA IA. Eight artefacts dating to the transition from NBA IB to NBA II and 57 artefacts dating to NBA II were also sampled for this investigation at the National Museum of Denmark, Copenhagen, with a view to exploring further developments leading to the peak of the NBA around 1400 BC.

The study then rests on a total of 543 analyses. Most of these artefacts were measured for trace elemental concentrations as well as lead isotope ratios (ten samples were only inspected by metallographic examination). This considerably enlarged dataset enables a long-term perspective on metal importation, use patterns, preferences, and structural correlations with known trading networks and societal change.

**Fig 1:**
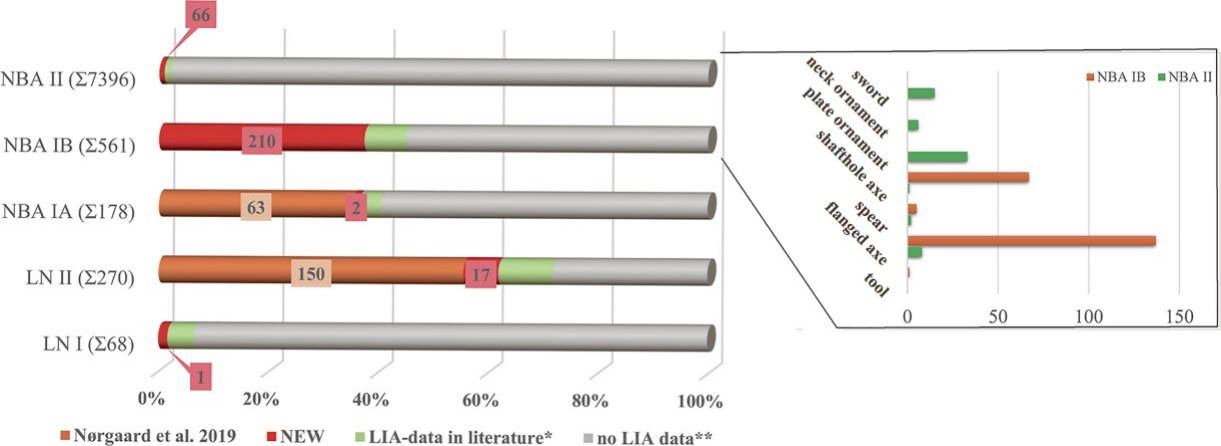
Analysed objects using lead isotope analysis (LIA) per period from the Neolithic to the established Bronze Age in southern Scandinavia. The number of analyses presented in this study is related to the published analyses [1,3,4,6–8,14,50,51] and given in percentages of the overall number of artefacts assumed for each period based on [17,48]. For NBA IB and NBA II, the chief focus of this article, the distribution of the artefact categories analysed within the present study is shown in the separate diagram.

The most widely distributed artefacts in the early period of metal use in southern Scandinavia comprise mainly weapons and tools (notably axes) (Fig 1). From the onset of the NBA, c. 1600 BC, to its full bloom after c. 1500 BC, artefact repertoire and refinement expanded greatly. At 1700 BC, spearheads come on the scene, followed by the first swords c. 1600 BC [4]. Typological classification has revealed distinct artefact styles with specific distribution areas at regional and supraregional levels [4,48]. Additionally, decorative styles and technological traditions are region-specific and allow us to distinguish between locally produced and imported objects [47,49]. Consequently, artefacts of a specific characteristic style, or their technological attributes, can be related to distinct regions, and local productions can easily be distinguished from foreign styles. In several cases, especially 2100–1600 BC, the local Scandinavian metalwork is sensitive to foreign styles, which have been creatively translated into items that fit local tastes, also easily identified. After c. 1600 BC, the full repertoire of metal objects in southern Scandinavia testifies to a very distinct Nordic style. In short, this new large dataset, used in conjunction with our knowledge of mobility patterns in general, allows us to track the major trade networks that linked southern Scandinavia with other regions in Europe. At its peak, this early trade became the scaffolding of the flourishing metal industry in the crucial later Middle Bronze Age of the fourteenth and thirteenth centuries BC.

Pre-Bronze Age samples are mainly from flat axes dating from the Earlier Neolithic to LN I [4] in the Danish region. Their copper sources are crucial for characterising the earliest stages of metal use [52,53], during which flint technologies thrived. Most Neolithic metal artefacts, including the axes discussed below, are likely European Chalcolithic imports or remelts (copies). This early metalwork and its regions of origin can illuminate cultural linkages before the first increase in metal use during LN II from c. 2100 BC onwards. The cornerstone of the study is a profound knowledge of the stylistic affiliations of artefacts to specific cultural and chronological groups. This has the potential, ultimately enable us to differentiate between geochemically largely similar metal sources. Thus, for the following periods, 2100–1300 BC, several artefact categories–axes, bladed weapons, ornaments–have been investigated, and within these categories, different stylistic types. Particularly regarding the sizeable number of axes, groups of foreign-style and local-style specimens serve for comparing the imported foreign artefacts’ chemical patterns with those of local production. This considerably aids the classification and provenance discussion of the raw material for the local production. In addition, our previous study [1] succeeded in demonstrating that for the early period of metalworking, at least until 1600 BC, true ingots did not exist. Instead, metal entered southern Scandinavia in the shape of ready-made artefacts, especially rings and axes.

## 3. Methods

### 3.1 Sampling and preparation of samples

Most of the samples were obtained from the sample archive of the Stuttgarter Metallanalysen project (SAM), acquired between 1954 and 1974 [54–56]. Our use of sample material remaining from Carl Cullberg´s investigations between 1959 and 1962 [57] was our response to the ethical responsibility of preserving our cultural assets for posterity and avoiding destructive investigations where possible. Thus 75% of the data presented here derives from reusing samples from the collection stored at the Curt Engelhorn Zentrum Archäometrie (CEZA) in Mannheim. Seventy-seven additional samples were taken in the years 2016–2017 from artefacts stored at the National Museum of Denmark in Copenhagen. These new specimens consist of drill-shavings. Sampling was performed with a 1 mm carbon-free steel drill in two steps: first, the patina was removed and collected; then, the drill-shavings from 1–2 mm deep drillings were collected. In preparation for trace element analysis using EDXRF, the patina within the stored samples was, if necessary, carefully removed under a microscope.

The prepared samples were processed through three methodologies, as described below. The entire research process was supported by suitable descriptive statistics. First, plots of lead isotope ratios and trace elemental concentrations, in combination with the crucial typochronology of the archaeological artefacts, underline the analytical outcomes of dispersions and groupings. Second, basic interpretations of the most likely provenance of the compositional copper types in question are discussed for the known variation ranges of copper ores (see section four, Results and Discussion). Finally, further theory-informed interpretation proceeds with the proposed provenance regions and their implications for how we may understand the rich NBA, its emergence, and its consolidation.

### 3.2 Trace element composition via EDXRF

The elements Fe, Co, Ni, Zn, As, Se, Ag, Sn, Sb, Te, Au, Pb and Bi were measured using an energy dispersive X-ray fluorescence (EDXRF) device (ARL Quant X, Thermo Scientific) at the CEZA in Mannheim, Germany. The samples were placed within the device using a twenty-position sample changer, which is especially useful for drilled samples. The samples were measured in two exposures of 600 seconds each [58]. Two reference materials obtained from the Bundesanstalt für Materialprüfung in Berlin (BAM211 and BAM376) were included in each run. Detection limits are, as indicated in the database (see S2 Table), 0.05% for Fe, around 0.01% for Co, Ni, and As, and about 0.005% for Ag, Sb, Sn, Au, Pb, and Bi. Mn, Cd, Se, and Te were measured, but were below 0.005% in all samples. Zn was below the detection limit of 0.1% in all samples and as such is not listed in the supplementary data (S2 Table).

Compared with the SAM analyses [54–56] we did not include sulphur for two reasons: First, the detection limits for the measurement of sulphur with XRF in air is rather high and second, the concentration of sulphur does not provide any information on the provenance of the copper. It does not even allow an unequivocal distinction between oxidic and sulphidic ores, because the high affinity of copper for sulphur tends to collect sulphur in the smelted copper. Since oxidic ores mostly derive from oxidation of sulphidic ores they often contain minor amounts of remaining sulphides. Accordingly, the sulphur content in copper is merely an indication of the quality of refining, which was not the subject of this study.

### 3.3 Cluster analysis

**Fig 2:**
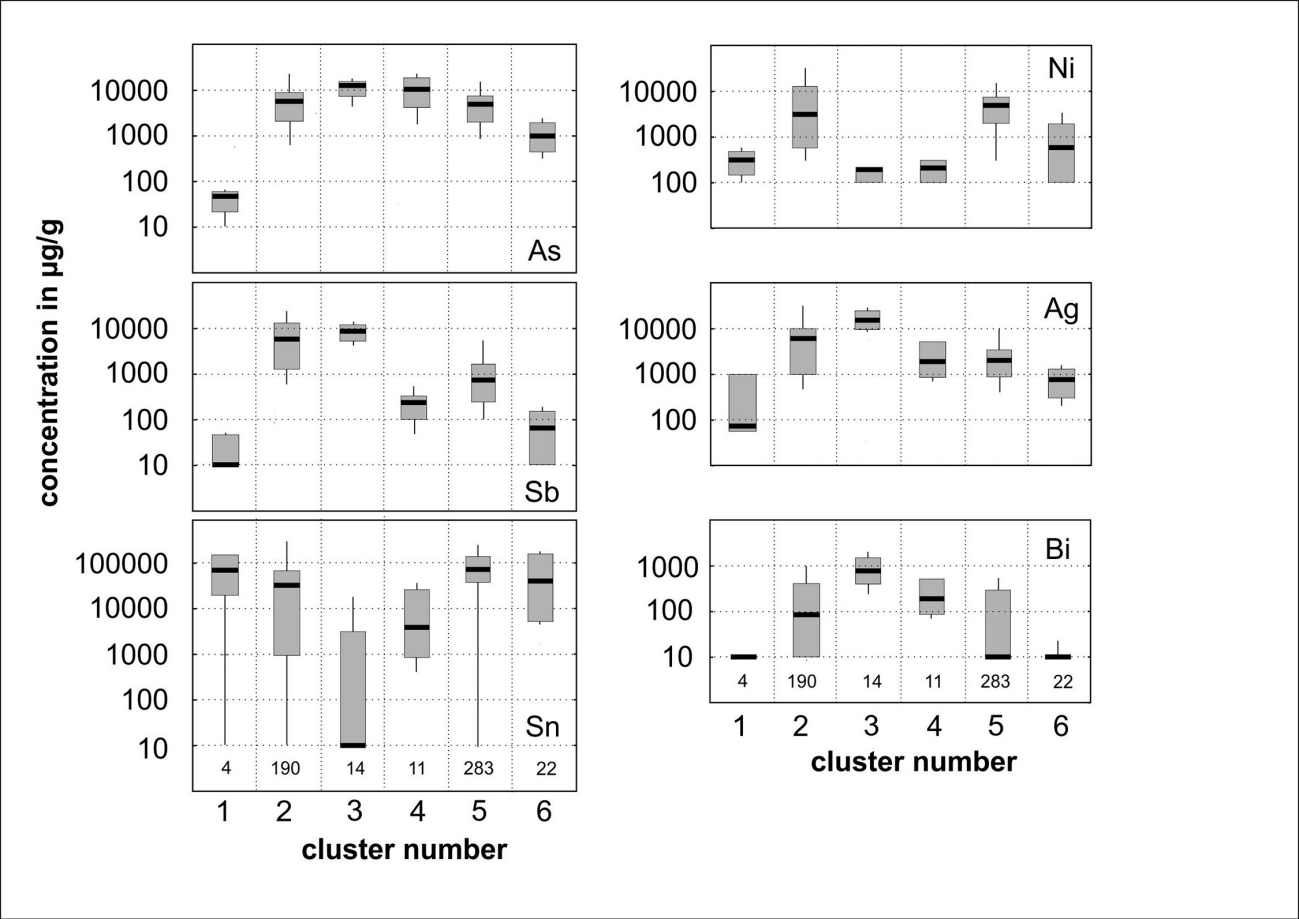
Compositional patterns of the major clusters identified among 533 samples from objects of the Nordic Bronze Age. Plotted are the medians (black), the interpercentiles (10 and 90%) and the total ranges (minimum and maximum). Clusters #7 to #11 contained only one or two samples and are considered as outliers or were combined with a larger cluster, which is compositionally most similar. Only logarithmic concentrations of As, Sb, Ni, Ag, and Bi were used for classification with cluster analysis. The ranges of tin concentrations are merely shown to demonstrate that certain chemical groups (C#3 and C#4) were not intentionally alloyed with tin.

For this study, average-link cluster analysis based on logarithmic concentrations of arsenic (As), antimony (Sb), silver (Ag), nickel (Ni) and bismuth (Bi) was performed using samples from 533 copper and bronze objects (the ten samples used for metallographic examination were not included). This methodology was chosen to enable sorting of the large dataset according to trace element patterns. In contrast, more recently suggested methodologies, based on the presence/absence of trace elements and an arbitrary threshold concentration of 0.1% [59,60], are less suited to data that covers concentration ranges of several orders of magnitude (Fig 2). On the one hand, one loses the possibility to identify compositional groups that may exist and can be recognised by frequency analysis, for example if the histogram of one element shows two or more peaks. On the other hand, one risks dividing analytical data that clearly belong together. A case in point is the composition of the well-known East Alpine copper, which is characterised by correlated concentrations of arsenic and nickel between 0.01 and 1% [40].

A new classification of the complete dataset was performed independently of the previous one. The previous published cluster analysis contained only a part of the data and concentrated on the early phases of continuous metal use 2100–1600 BC [1]. The new run, covering analyses from artefacts dating from 3800 BC to 1300 BC, resulted in eleven compositional groups, each displaying their own characteristics (Table 2). Compositional differences from the previous cluster analysis of the smaller dataset (Table 3) are discussed below. Within the new classification, both types of Ni-bearing fahlores are combined into cluster C#2 (with one artefact remaining in a separate group, C#11). This similarity in the new classification illustrates the large overlap between the two Ni-bearing fahlore groups, thus supporting the assumption that both might originate from the same region and may in fact represent different mines or shafts in the same source region [1]. Furthermore, the separation between relatively pure and low-impurity copper has become more pronounced, as clusters C#6 and C#5 demonstrate. The majority of the material (53%, 284 samples) is allocated to cluster C#5, which prompted the team to take a closer look at this compositional group. In a second run, cluster C#5 was split up into eleven subclusters. Parameters exist that help to decide which number of clusters provides an optimal grouping: within-cluster variation should tend towards a minimum, and between-cluster variation towards a maximum. Some intuition by the researcher is still called for, however, as cluster analysis is a heuristic method and can be considered an aid to finding structure among a large number of data. The hard test of the usefulness of a particular classification is, of course, that duplicate analyses of the same object should be classified in the same cluster. Accordingly, the results of cluster analysis need further refining, either by discriminant analysis calculating the probability of each sample belonging to the cluster it is assigned to, or simply by archaeological reasoning concerning, for example, the context of the artefacts.

**Table 2:** Elemental compositions (average) of the ten major clusters discussed in this study.

	[wt%]	Cu	Ni	As	Ag	Sn	Sb	Pb	Bi
**Cluster #1**	Average	93	0.03	0.00	0.01	6.93	0.002	0.002	0.001
(4 sample)	Median	92	0.03	0.01	0.01	8.15	0.001	0.001	0.001
**Cluster #2**	Average	94	0.52	0.56	0.62	3.46	0.605	0.094	0.014
(190 sample)	Median	94	0.33	0.48	0.69	2.80	0.552	0.010	0.009
**Cluster #3**	Average	96	0.02	1.41	1.32	0.122	0.928	0.016	0.131
(14 sample)	Median	96	0.02	1.43	1.21	0.001	0.924	0.008	0.119
**Cluster #4**	Average	99	0.02	0.95	0.03	0.002	0.020	0.027	0.004
(11 sample)	Median	99	0.02	1.12	0.02	0.001	0.018	0.017	0.002
**Cluster #5**	Average	91	0.48	0.46	0.02	8.11	0.089	0.156	0.002
(285 sample)	Median	91	0.48	0.44	0.02	7.90	0.069	0.051	0.001
**Cluster #6**	Average	91	0.09	0.11	0.01	9.17	0.008	0.009	0.001
(22 sample)	Median	91	0.06	0.10	0.01	8.80	0.007	0.006	0.001
**Cluster #7**	Average	91	2.29	2.41	0.20	3.05	0.796	0.131	0.004
(2 sample)	Median	91	2.29	2.41	0.20	3.05	0.796	0.131	0.004
**Cluster #8**	Average	97	0.83	0.02	0.96	0.001	0.837	0.082	0.001
(1 sample)	Median	97	0.83	0.02	0.96	0.001	0.837	0.082	0.001
**Cluster #9**	Average	93	0.02	0.02	0.42	6.55	0.014	0.001	0.002
(2 sample)	Median	93	0.02	0.02	0.42	6.55	0.014	0.001	0.002
**Cluster #10**	Average	99	0.01	0.63	0.72	0.001	0.001	0.004	0.001
(2 sample)	Median	99	0.01	0.63	0.72	0.001	0.001	0.004	0.001

C#11 consists of a single artefact with a trace element pattern similar to C#5. The single sample is integrated in the sample number stated at Cluster #5.

**Table 3:** Structure of the clustered data with numbers of artefacts/members, based on the average-link cluster analysis with logarithmic concentrations of arsenic (As), antimony (Sb), silver (Ag), nickel (Ni) and bismuth (Bi).

2020 (533 samples)	Members	2019 (422 samples)	Members
1	4	1	2
2*	190	10*	86
3*	14	12*	12
4	11	5+6+7	9
5+11	285	4	231
6	22	8	24
7*	2	11*	53
8	1	13	1
9	2	2	3
10	2	3	1

* fahlore (high impurity copper).

Also included are the results of the previous cluster analysis of the smaller dataset [1].

### 3.4 Lead isotope analysis using MC-ICP-MS

For the measurement of the lead isotope ratios of ^208^Pb/^206^Pb, ^207^Pb/^206^Pb and ^206^Pb^/204^Pb, performed by multiple-collector inductively coupled plasma mass spectrometry (MC-ICP-MS) at CEZA in Mannheim, Germany, a Thermo Scientific Neptune Plus multi-collector mass spectrometer was used. The samples were rinsed with dilute HNO_3_ to remove any surface contamination, then dissolved in half-concentrated HNO_3_ in an ultrasonic bath (70°C) for several hours. Insoluble residues were removed by decantation from the resulting solution, which then was diluted with deionised water [25]. Columns were prepared with PRE-filter resin and Sr-resin and were preconditioned with 500 μ 3N HNO_3_ before the solution was added. In four steps, the matrix was first eluted using HNO_3_, and then the Pb was eluted using HCl. After drying (48h), 50 ng/g thallium was added to the sample solution to correct internal mass fractionation [61]. Solutions were prepared aiming at 100 ng ml^-1^ lead and 50 ng ml^-1^ thallium. The sample-holder of the Thermo Scientific Neptune Plus multi-collector MC-ICP-MS offers space for 48 samples. Standard reference sample NIST SRM 981 was measured after each eight samples to check for instrumental drift and guarantee high-level precision and accuracy. The measured values of NIST SRM 981 compare favourably with those reported in the literature (Table 4). The ratios of ^208^Pb/^206^Pb, ^207^Pb/^206^Pb, and ^206^Pb/^204^Pb, were measured, while the ^208^Pb/^204^Pb, ^207^Pb/^204^Pb ratios were calculated based on the measured ratios.

**Table 4:** Measured values of NIST SRM 981 from different literature sources compared with those obtained in this study.

Galer and Abouchami (1998)	36.7219	44	15.4963	16	16.9405	15	2.16771	10	0.91475	35
Hirata (1996)	36.68	210	*15*.*4856*	* *	16.9311	90	2.16636	82	0.914623	37
Rehkämper and Halliday (1998)	36.6969	128	15.4912	51	16.9364	55	2.16677	14	0.914685	49
Rehkämper and Mezger (2000)	36.7	23	15.49	17	16.9366	29	2.16691	29	0.91459	13
Todt et al. (1996)	36.7006	34	15.4891	9	16.9356	7	2.16701	13	0.914585	4
Thirlwall (2000)	36.7228	80	15.4956	26	16.9409	22	2.1677	21	0.91469	7
White et al. (2000)	36.6825	78	15.4899	39	16.9467	76	2.1646	8	*0*.*91404*	* *
This study (n = 339)	36.684	11	15.486	3	16.933	3	2.1664	3	0.91456	9

Analytical errors (2 sigma) refer to the least significant digits and results shown in italics were calculated from the data given in the original publication [62–68].

## 4. Results and discussion

### 4.1 Copper to Neolithic southern Scandinavia

Thirteen flat axes and an arm spiral found in Denmark, typologically dating to the first part of the Earlier Neolithic (early Funnel-Beaker culture 3800–3500 BC) [52] or to LN I (2350–2100 BC), were included in the investigation. The decision to include pre-Bronze Age metalwork concurred with the aim of identifying those exchange networks that were the first to introduce copper artefacts, and to a certain extent also metallurgical practice, to southern Scandinavia. Copper in Nordic Neolithic contexts has long fascinated Scandinavian archaeology [13,14,52], and some of the results presented below are re-analyses of earlier published data [14], namely #3, #35–36, #42 and #303.

**Fig 3:**
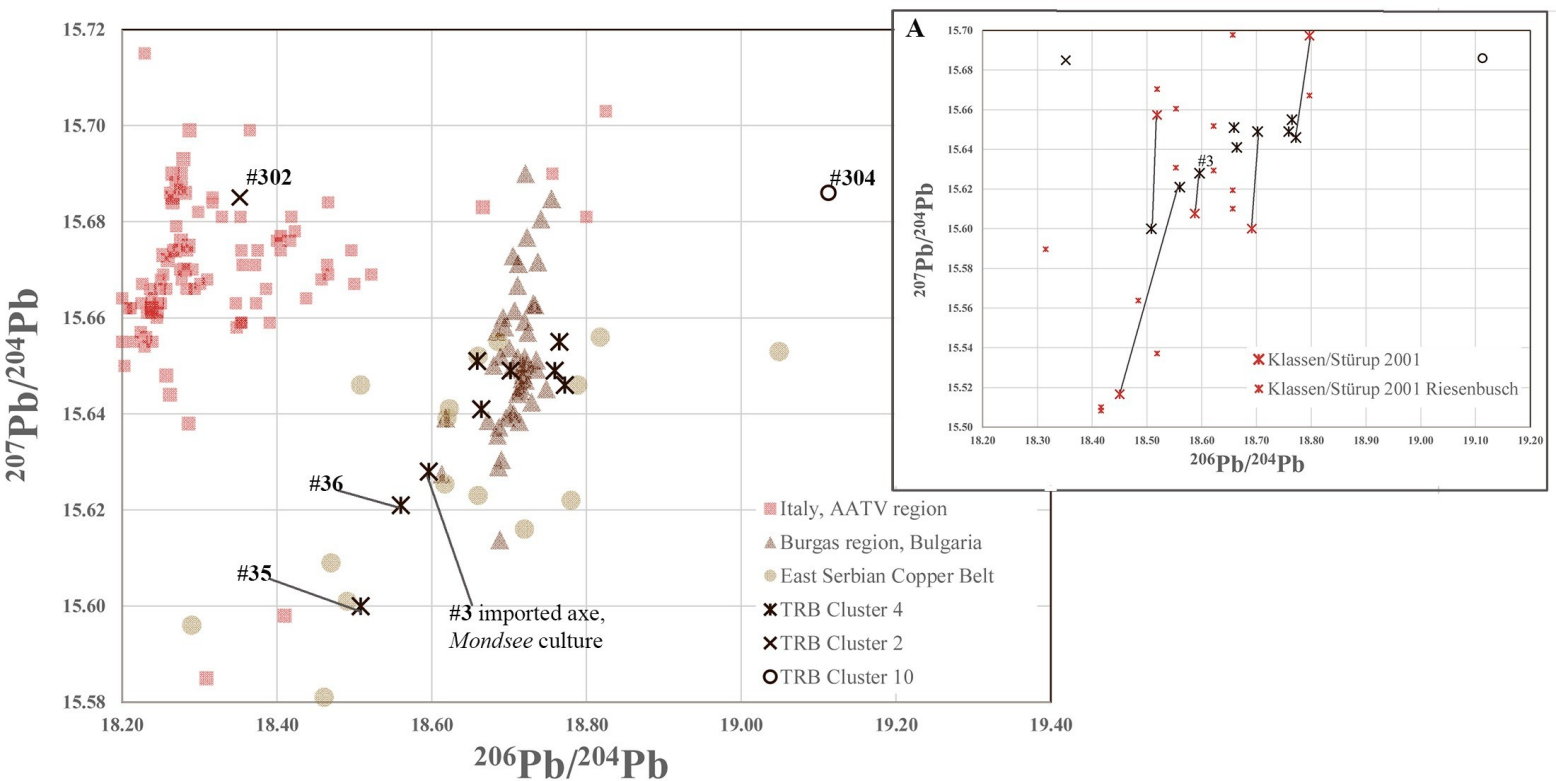
^206^Pb^/204^Pb and ^207^Pb/^204^Pb isotope plot of the copper flat axes discussed, with comparative plot (A) indicating the difference of measurements between this study and published ones [14], obviously problematic in their ^207^Pb/^204^Pb ratios. The ore data are from: Italy AATV mining region [32,73–75], Serbian copper deposits [72,76], Bulgarian copper deposits [26,33].

The nine copper flat axes of Bygholm type belonging to the early Funnel-Beaker period are all made of medium-arsenical copper with an average arsenic content of 0.9% and Ni = Ag = Sb around 0.02%. This copper has a characteristically low impurity signature, apart from the arsenic. The flat axe and arm spiral from the Søby Hede hoard (#42–43) both belong in this early group. Evaluation of lead isotope ratios of five reanalysed flat axes and four new analyses supports the general opinion that this so far unprovenanced Riesebusch-Mondsee copper [14,69,70] cannot be of East Alpine origin (Fig 3). Rather, the geographical distribution of such relatively pure arsenical copper suggests that it derived from south-east Europe, supported by the dagger being a new type of metal weapon in the flourishing Chalcolithic environment of the fourth millennium BC [69,71]. The dagger blade from the Danish Bygholm hoard exemplifies this. Indeed, Frank and Pernicka [69] observed that this copper type’s purity corresponds with that of south-east European Chalcolithic heavy implements (e.g. shaft-hole axes), which mainly relate to the copper deposit of Majdanpek in Serbia. The measured ^206^Pb^/204^Pb lead isotope ratio of 18.56 and the ^207^Pb/^204^Pb ratio of 15.621 from the ovoid, tongue-shaped flat axe from Viborg #36 are comparable with the known values of the Ai Bunar mine at Stara Zagora in Bulgaria [see 26]. The slightly lower values of the thick-butted flat axe from Slusegård #35 are within the range of both the Chalcolithic copper mines near Stara Zagora (Ai Bunar) and Burgas (Medni Rid), as well as the contemporary mines in the East Serbian Copper Belt [see 72]. The axe from Moesgaard #3 is probably an import via Austria’s Mondsee group, a branch of south-east European Chalcolithic groups [52]. Six other flat axes likely derive from Serbian ores, based on the comparative values from the Serbian region [72].

To sum up, the new results presented above differ from those established by previous research [14,52]. The main reason is the much-improved precision and accuracy of the measurements with MC-ICP-MS, which produce results comparing well with analyses performed with thermal ionisation mass spectrometry (TIMS) on the Bulgarian ores [26]. We therefore suggest that copper flat axes and copper trinkets made of south-east European ore travelled from or via the Mondsee culture in Austria to southern Scandinavia early in the Funnel-Beaker period. To explain the relatively higher arsenic contents, it is very likely, as Frank and Pernicka suggest [69], that a new technology had been introduced–namely the intentional addition of arsenic in the form of speiss (iron arsenides, [77]) to the already available copper. The Søby Hede hoard (#42–43) strengthens this supposition: the six times higher arsenic content of the axe (1.17%) in relation to the arm spiral (0.20%) could well be intentional. Although such a process has only been identified in Iran during the final fourth millennium BC [77,78], enrichment with arsenic may well have been invented earlier, thus giving rise to the phenomenon of arsenical copper as the dominant metal type of the rich Chalcolithic of the fourth millennium BC in south-east Europe. This hypothesis is now corroborated by the suite of Funnel-Beaker flat axes isotopically matching Bulgarian and Serbian copper ores. In conclusion, the copper from which these early items were made–including the hitherto unprovenanced Riesebusch-Mondsee copper–was most likely mined in Serbia and Bulgaria in south-eastern Europe.

In the late group of axes–tentatively dated to the LN I Bell-Beaker culture c. 2350–2100 BC [52,56]–the new metal analyses indicate an origin completely different from the axes of Riesebusch-Mondsee copper discussed above, and possibly also a wider chronological range than strictly “Bell-Beaker.” The axe from Kjelstrup in south Jutland #304 consists of high-impurity copper with Sb = Ag>As, a dilute antimonial copper especially abundant in Bosnia in the late fourth and third millennia BC [55], a region where antimony-bearing minerals are also abundant [79]. Antimonial copper with exceptionally high concentrations of antimony of more than 10% are occasionally found in the Late Bronze Age of western Hungary near Velem. St. Vid [80] and also in adjacent Lower Austria, albeit with lower antimony concentrations up to 3.5% and with lead isotope ratios consistent with the Slovakian Ore Mountains [81]. Isotopically this axe is consistent with fahlores from the Valais in Switzerland, but antimonial copper seems rare there. The other axe #302 has arsenic and silver as major impurities, a ^206^Pb^/204^Pb ratio of 19.113, and a ^207^Pb/^204^Pb ratio of 15.686, which is found in the south-east Alpine regions of the Alto Adige, Trentino and Veneto (henceforth AATV). The flat axes #44 and #45 have extreme values of 23.845/22.547 ^206^Pb^/204^Pb and 1.6195/1.712 ^208^Pb/^206^Pb, indicating an ore deposit with highly radiogenic lead. Similar values have been recorded in Rudna Glava and Crnajka near Majdanpek in Serbia [72], but also in eastern Bulgaria, where Chalcolithic copper smelting has been attested [82].

To sum up. The group of later flat axes are typologically much more varied and often smaller than the thick-butted trapezoidal flat axes of the Bygholm type [4]. The dataset presented here corroborates the observation that the varied group of non-Bygholm axes shows much diversity in copper composition. Earlier research uses the term “Dutch Bell-Beaker copper” (e.g. [4], which might well originate from several different sources, including south-east Alpine ores), but in fact two of the axes analysed (#44, #45) are neither an analytical match for the Riesebusch-Mondsee copper nor seem to fall within the known variation among LN I Bell-Beaker-affiliated artefacts. An explanation could be that they may have arrived in Scandinavia much earlier, sometime during the later Earlier Neolithic or the Younger Neolithic (3500–2400 BC). If so, copper flat axes in some measure reached southern Scandinavia in the long metal-poor period between the two early peaks in copper appropriation. Future typological and context studies may shed more light on this material.

### 4.2 Metal growth at the turn to the Bronze Age: LN II (2100–1700 BC)

**Fig 4:**
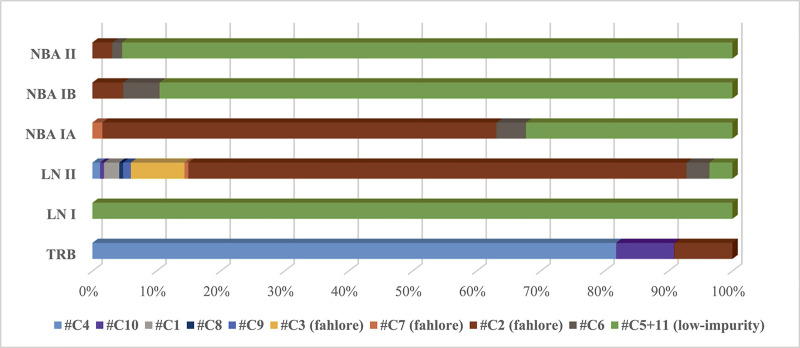
Distribution of the different copper types (clusters: For definition see text) in time. The largest variety in compositional groups is observed for the LN II period. In contrast, in the developed Bronze Age, NBA IB-II, only two different compositional types of low-impurity copper were used, and only minor amounts of fahlore copper.

**Fig 5:**
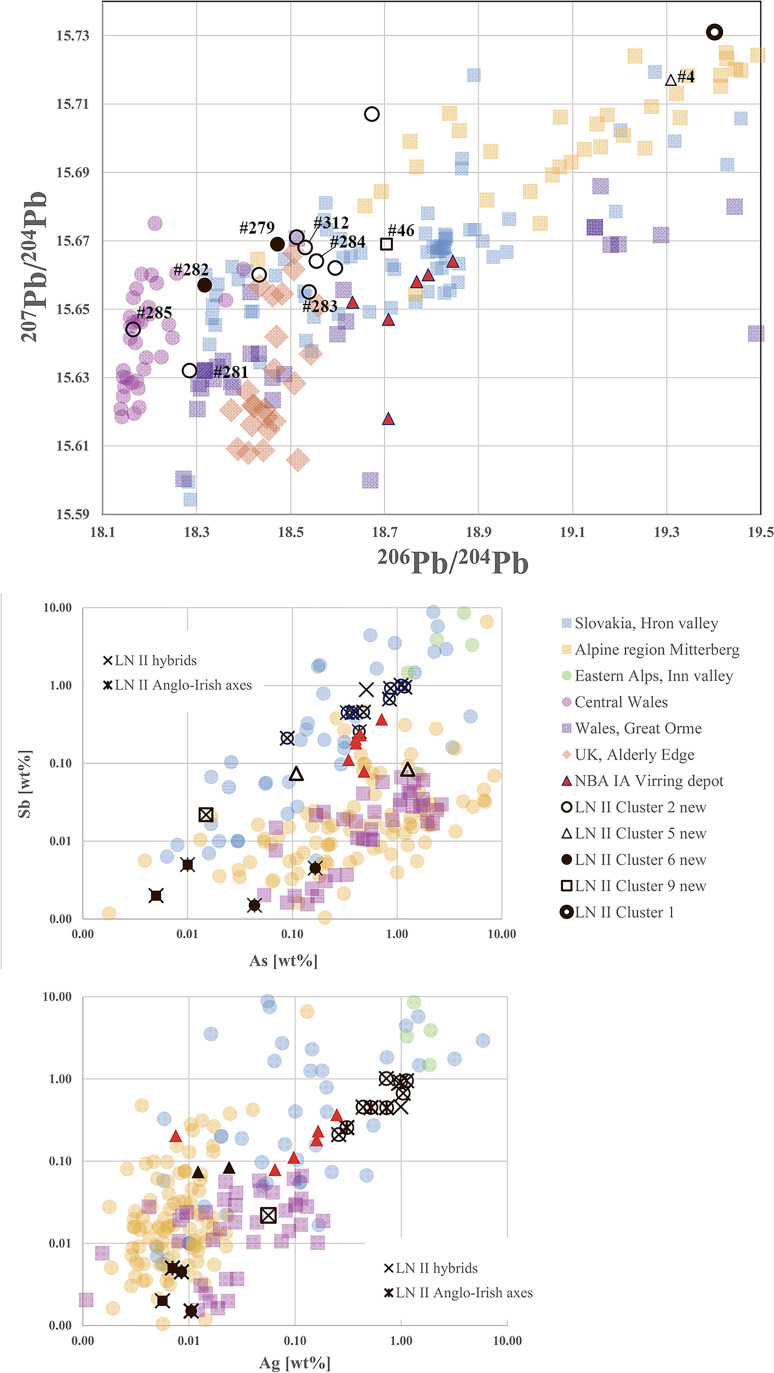
Chemical and isotopic evaluation of LN II and NBA IA samples in this study. The ^206^Pb^/204^Pb and ^207^Pb/^204^Pb diagram compares the LN II artefacts and the objects from the NBA IA Virring deposit, Jutland, with the discussed copper deposits. The trace elemental comparison of Sb-As and Sb–Ag allows for identification of British/Welsh metal and highlights the probability of use of Slovakian fahlore copper. For comparing elemental with classificatory data, the Anglo-Irish axes and hybrid forms are highlighted with the respective symbols shown in the Sb-As and Sb–Ag data-plot, placed on top of the cluster symbols. The ore data are from: Mitterberg ore district [35], Hron Valley, Slovakia [27,28], Inn Valley and Buchberg, Alpine region [29,30], Great Orme mining region, Wales [36–39,83], Alderley Edge mining region, Britain [39,83], central Wales mining region [39,84].

The first significant metal growth in southern Scandinavia took place in the LN II period, testifying to a fully metal-using society in southern Scandinavia contemporary with the Early Bronze Age in Europe. Our recent publications have demonstrated that in LN II Scandinavians were keenly trading metals that originated in the British Isles, the Slovakian Ore Mountains, and the East Alpine Inn valley. Several groups of fahlore-type copper predominate among these imported metals (Fig 4). A western maritime route led from southern Scandinavia to landing places in southern England, while an eastern maritime and riverine route steered across the Baltic Sea and continued upstream along rivers to the heart of the Únětice culture, a key crossroads thriving on the trade in Alpine and Slovakian copper to Scandinavia [1,17]. The additional twenty analyses from LN II align with these results (Fig 5). They corroborate the Nordic practice of mixing artefact metal of various origin into the production of desired local-style artefacts. Notably in LN II, British high-tin bronze flat axes–imported to southern Scandinavia–lent strength and golden colour to local axes. In several cases, the British design of trapezoidal shape combined with surface-covering ornamentation was translated into local taste, resulting in so-called “British derivatives”.

Three typical *British bronze flat axes* from the hoards of Gallemose, Selchausdal and Store-Heddinge (#46, #279, #282) reveal the typically British low-impurity copper with high tin content. Another trapezoidal, though plain axe from Brokbjerg (#4) and a British wave-decorated bronze flat axe from Beddinge, Scania (#310) with a similar composition may also be imports. Whereas two of the five named axes confirm their import status with isotopic signatures consistent with Welsh and British ores such as Alderley Edge (#279, #282), the others show distinctly deviating isotope signatures. Five of the newly examined LN II axes of typical local manufacture were made of high-impurity copper on a low tin basis, which is standard for the period (#1, #33–34, #39, #41; S2 Table). Two fall within the range of the Slovakian Ore Mountains. In contrast, six axes in the same copper group (C#2) have medium-to-high tin values. They are typologically British derivatives, with a hybrid appearance testifying to local recasting (#280–281, #283–285, #309). Such hybrids often occur together with genuine British axes in hoards (e.g. Selchausdal and Store-Heddinge), pointing to locally based use of British high-tin axes to enrich local products aesthetically as well as to achieve use-related properties. In short, the hybrids incorporate stylistic elements from imported British axes with intrinsic Nordic features in their form and decoration. Their isotopic signatures are correspondingly diverse, ranging from 18.16 to 18.7 and from 15.63 to 15.67 for ^206^Pb/^204^Pb and for ^207^Pb/^204^Pb. British, Slovakian, and possibly also Swiss copper ores [85] plot within these ranges (Fig 5). Some hybrids (like #283–284, #311) are, in terms of their recast metal, isotopically still very close to their British antecedents, while others are somewhat more remote: the British derivatives from Flenstofte (#281) and Store-Heddinge (#285) are isotopically comparable to the signatures known from the Great Orme and central Welsh mines, while their trace element composition aligns with Slovakian fahlore-type copper (see Fig 5). The local smith clearly mixed the material to achieve or preserve desirable qualities in the local casts, whether these were colour, decoration, or strength. Many hybrid axes appear to have been made from a mixture of high-tin bronze imported in the form of the well-known British bronze flat axes and the frequently used Slovakian fahlore copper, probably also to accommodate the required amount of metal. The resulting cast would be a high-to-medium-tin bronze with mixed geochemical signatures, a phenomenon quite frequent in the otherwise tin-poor Scandinavian Late Neolithic metalwork.

### 4.3 Metal growth at the turn to the Bronze Age: NBA IA (1700–1600 BC)

Metal growth continued on a moderate scale throughout NBA IA, revealing a consolidated metal use in southern Scandinavia contemporary with the ending of Europe’s Early Bronze Age. Our previous work highlighted the first arrival in NBA IA of a distinct type of non-fahlore copper, namely a low-impurity copper probably smelted from copper ores rich in chalcopyrite. According to the new cluster analysis of the complete dataset, characteristic low-impurity copper (clusters C#6 and C#5+11) only amounts to about 36% of the copper that reached Scandinavia in NBA IA (compare [1]). This “chalcopyrite copper” supplemented the still predominant fahlore coppers (C#2, C#7). Various examples showed that mixing of both copper types for local production is very likely [1], and the large variety in the trace elemental composition of the NBA IA C#5 and C#2 bronzes highlights this (see S2 Table). Various metal types arrived in Scandinavia to form the foundation of the first full tin-bronze metalworking. Copper from British ores and especially from the Slovakian Ore Mountains become phased out in the NBA IA: at this point, it seems that a re-arrangement of metal-driven trading occurred.

The two new analyses dating from NBA IA are consistent with these results. With these extra analyses (#286–287), the Virring weapons hoard is now fully analysed. This deposit holds a special position within the study of the Nordic Early Bronze Age, because it includes three small locally made spearheads (Torsted type) and a large spearhead of central European type [4], accompanied by a long dagger and two local flanged axes with semicircular cutting-edge–a style fashionable elsewhere in Europe around 1700–1600 BC. The spearhead is a military innovation, appearing in central Europe and in southern Scandinavia c. 1700 BC. These four spearheads are probably made of copper from the same metal source, as trace element and isotope signatures indicate (Fig 5). One of the flanged axes has very similar signatures and is similarly made of high-impurity fahlore metal characteristic of the Slovakian Ore Mountains. The other axe, of Virring type [4], is also made of impurity-rich copper, though with a significantly lower ^207^Pb/^204^Pb ratio compared with Bulgarian and Serbian ores [26,33]. The compositional similarity of many of the Virring hoard objects may indicate that they were made from the same stock of metal, comparable to what is seen in LN II (above). The Virring-type axe, however, seems to have a different origin, supported by another Virring-type axe from Ejstrup Holm [1], which might point to the influence of south-east European sources.

### 4.4 Metal in the NBA 1600–1300 BC: low-impurity copper of chalcopyrite quality and its predominance from 1600 BC

From 1600 BC onwards, beginning with the initial rise of the Nordic Bronze Age in NBA IB, the preceding diversity of copper types (clusters) in southern Scandinavia came to an end. This new situation is distinct in the dataset, and coincides with large amounts of imported metal now circulating regionally. In total, 210 artefacts from the NBA IB (1600–1500 BC), eight artefacts from the earliest NBA II (1500–1450 BC) and 49 artefacts (with 58 samples) from the mature NBA II (1400–1300 BC) were analysed. The metal used for local production from NBA IB onwards is homogeneous in its trace element pattern, with nickel and arsenic as major impurities, and a tin content of around 7.8% in NBA IB and 9.4% in NBA II. Partly, a 1:1 correlation of nickel and arsenic was attested and may denote a specific copper source with gersdorffite, a nickel-arsenic sulphide mineral (NiAsS) that occurs, frequently as accessory mineral, in copper deposits in the East Alpine region (i.e. [86] for example at Mitterberg [1,35]). However, the Slovakian Ore Mountains, the Alto Adige, Trentino and Veneto in the Italian Alps of South Tyrol (AATV) and the Great Orme mining area in Wales show a similar relationship of nickel and arsenic [36,87], became available after 1600–1500 BC [88], and were within reach of the Nordic zone. The striking uniformity and predominance of a copper with low impurity levels suggesting smelting of chalcopyrite ores–in use at this time over large parts of non-Mediterranean Europe–have long puzzled researchers [89,90].

Despite the remarkable uniformity of the trace element composition, the isotopic signatures are quite variable. This might indicate more than one copper source with correlated concentrations of nickel and arsenic in the trade, or alternatively a single copper deposit with large variations in lead isotope ratios due to the presence of uranium. In addition, inconsistencies between trace element composition groups and the respective isotope signatures might indicate mixing, especially if a convergence of values is recognisable. This long-standing conundrum of similar but different requires further investigation.

#### 4.4.1 Provenancing copper used for massive shaft-hole axes of Nordic type 1600–1500 BC

**Fig 6:**
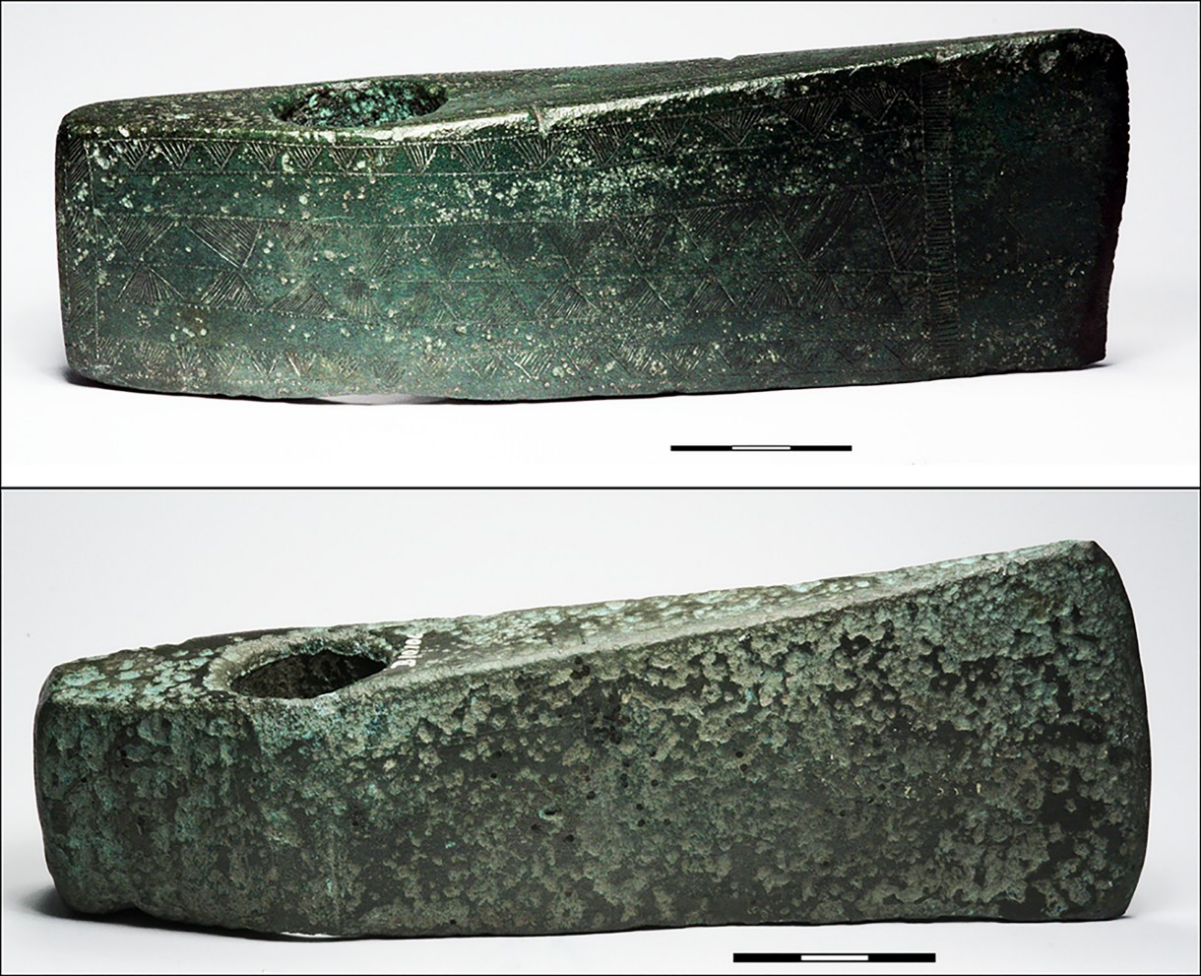
Decorated shaft-hole axes from an unknown find location, NM 541 (top) and Juellinge, Maribo Amt (NM B10106). Photo: Heide W. Nørgaard, by permission of the National Museum, Copenhagen.

**Fig 7:**
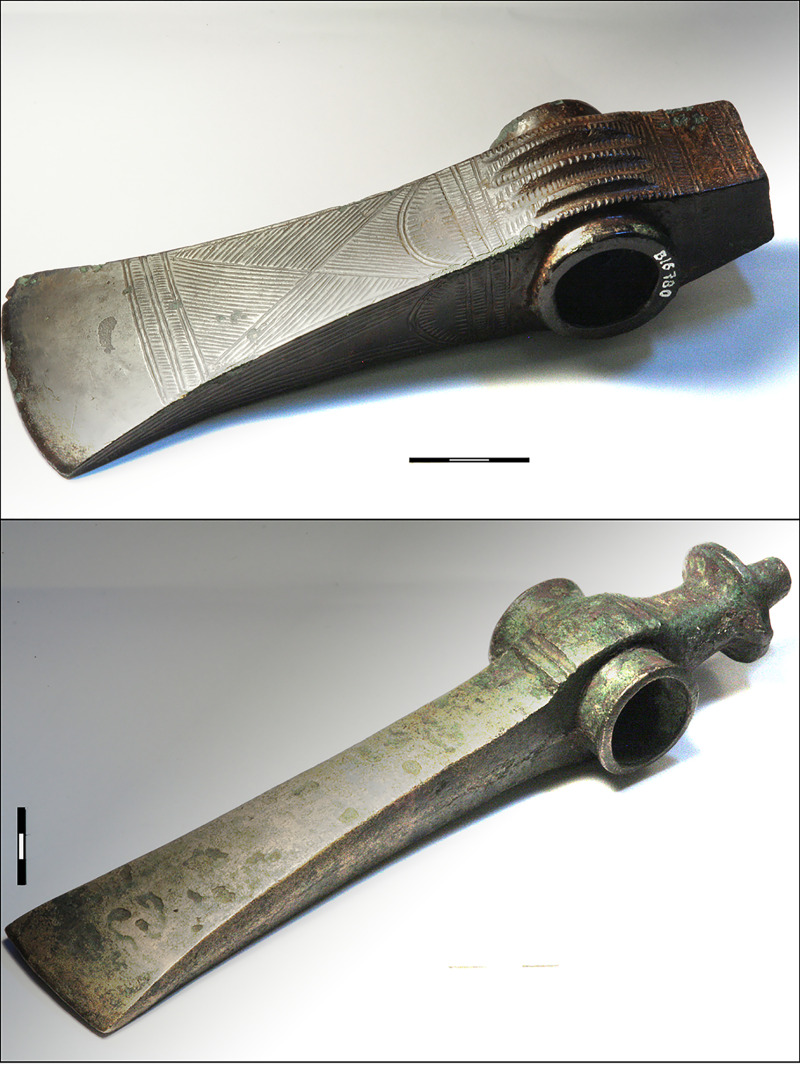
**Valsømagle-type axes are elegantly sculptured. Geometric decoration is rare, and the axe from Engemarken (top) is one of few artefacts with such a decoration.** Top: Decorated Valsømagle-type shaft-hole axe from Engemarken, Roskilde, København municipality (B16780). Bottom: The shaft-hole axe from Vesterå, Børglum, Hjørring municipality (NM 19176), is of south-east European style and can probably be dated to the beginning of the NBA II. Photo: Heide W. Nørgaard, by permission of the National Museum, Copenhagen.

From 1600 BC to c. 1500 BC, bronze shaft-hole axes make their remarkable entry in NBA material culture. Many kilograms of bronze went into the production of these heavy axes. Bronze shaft-hole axes divide into two types, the frequent Fårdrup type and the perhaps slightly later, more refined and less frequent Valsømagle type. Fårdrup-type axes (Fig 6) appear undecorated or, in half of all specimens, with more or less elaborate post-cast geometric decoration including the ogival V sign found on the *Hajdúsámson*-type swords and daggers [47]. These heavy axes are skeuomorphic translations into bronze of contemporaneous local stone shaft-hole axes, while often featuring decoration deriving from bronze axes, daggers and swords of the Carpathian basin [4,91–93]. Due to the striking similarity between stone-made and bronze-made shaft-hole axes, the bronze version is widely accepted as a Nordic product. The Valsømagle-type shaft-hole axes, in contrast, are mostly undecorated, but their form is elegantly sculptured. A few transitional axes with geometric decoration, however, integrate the characteristics of both axe categories (Fig 7). This might suggest a typochronological sequence from early Fårdrup to late Valsømagle, or even contemporaneity between them [4,94–96]. This question is pursued below through a large number of metal analyses. The few associated finds and broader associations of these axes seem to indicate at least parallel tracks during the approximately one hundred years’ duration of NBA IB [4].

The present study has analysed 67 shaft-hole axes from Denmark– 60 Fårdrup-type axes and seven Valsømagle-type axes–which corresponds to around 50% of known artefacts of these two types, thus increasing the previously available data for this group by a factor of ten [6,8]. We therefore foreground the question whether the uniformity in shape of the Fårdrup axes might indicate a single major copper resource, or whether these axes exhibit a higher degree of complexity in their provenance patterns. How do Fårdrup-type and Valsømagle-type axes relate to one another in terms of their copper?

**Fig 8:**
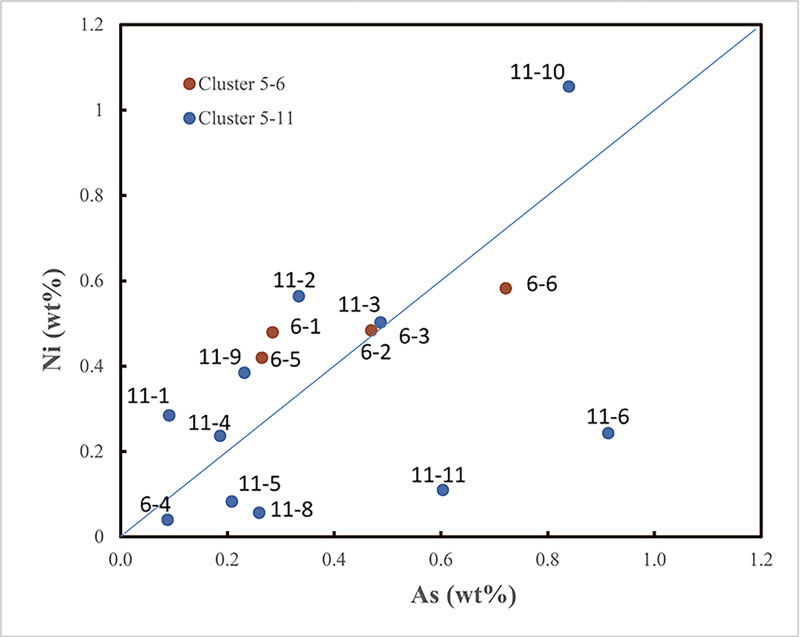
The subdivision of cluster C#5 is visible in the As/Ni diagram. The study presented used the subdivision into eleven subclusters as a guide to define these artefact groups with a significant Ni = As balance.

In ***step one***, the majority of the shaft-hole axes revealed a very homogeneous trace element pattern, with Ni = As and minor impurities of Ag and Sb between 0.01–0.3%, and an average of 8.3% Sn (characteristics of the subdivision cluster #C5–11–3, Fig 8).

**Fig 9:**
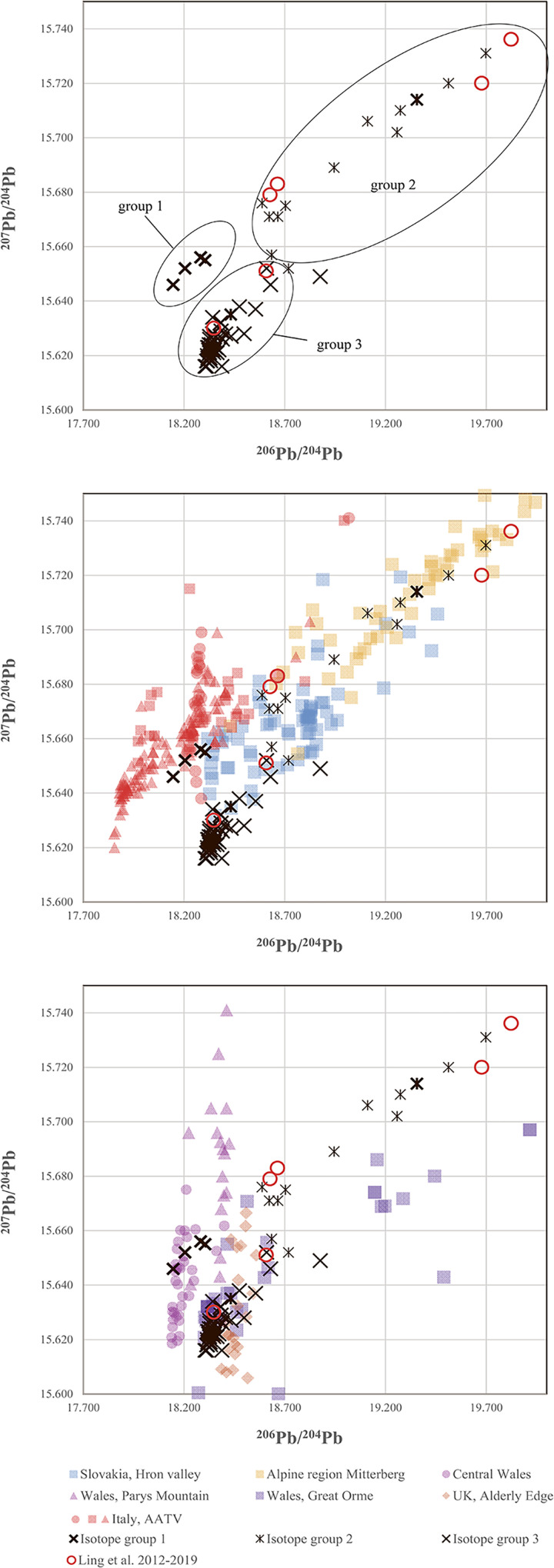
Diagrams of the ^206^Pb^/204^Pb and ^207^Pb/^204^Pb lead isotope ratios of the shaft-hole axes of NBA IB, including comparative reasons for the shaft-hole axes analysed by Ling and colleagues [6,8]. The three distinct groups are clearly recognisable (top). *Isotope group 1* matches the isotope ratios of the Italian Alps [68], both in the conventional diagram (not shown), lying in between ratios of 2.09–2.112 ^208^Pb/^206^Pb and 0.855–0.862 ^207^Pb/^206^Pb, and in the plot presented. *Isotope group 2* has a much larger variation, 2.03–2.083 ^208^Pb/^206^Pb, 0.81–0.843 ^207^Pb/^206^Pb and 18.58–19.69 ^206^Pb/^204^Pb, within the range of the Slovakian and East Alpine ores from Mitterberg. *Isotope group 3* shows a very dense concentration and does not exceed values of 18.555 ^206^Pb/^204^Pb and 15.638 ^207^Pb/^204^Pb, in line with British and Welsh ores. The ore data are from: Mitterberg ore district [35], Hron Valley, Slovakia [27,28], Inn Valley and Buchberg, Alpine region [29,30], Great Orme mining region, Wales [36–39,83], Alderley Edge mining region, Britain [39,83], central Wales mining region [39,84], Italy AATV mining region [32,73–75], Valais, Switzerland [31,85]. The analytical uncertainties are shown by the size of the symbols.

In ***step two***, lead isotope ratios suggested subdivisions. Fig 9 displays the ^206^Pb^/204^Pb, ^207^Pb/^204^Pb isotope ratios measured in the 67 Nordic shaft-hole axes. Three groups are identifiable, and it is notable that all three include both axe types. Isotope group 1 (four axes, 6%) fully matches the isotope ratios of the Italian Alps [75] in the conventional diagram (not shown) as well as in the ^206^Pb^/204^Pb vs ^207^Pb/^204^Pb plot. Isotope group 2 (14 axes, 21%) has a much larger variation of 2.03–2.08 for ^208^Pb/^206^Pb and 18.58–19.69 for ^206^Pb^/204^Pb, which extends to radiogenic lead with negative model ages. This group also includes two axes made of high-impurity metal (#27, #175). Within this range lie both the Slovakian and the East Alpine ores at Mitterberg [27,28,35]. Isotope group 3 (49 axes, 73%) discloses a very dense concentration and does not exceed 18.55 ^206^Pb^/204^Pb and 15.63 ^207^Pb/^204^Pb, which, in Europe, matches the English and Welsh mining areas, specifically the Great Orme mines [36,39,87].

**Fig 10:**
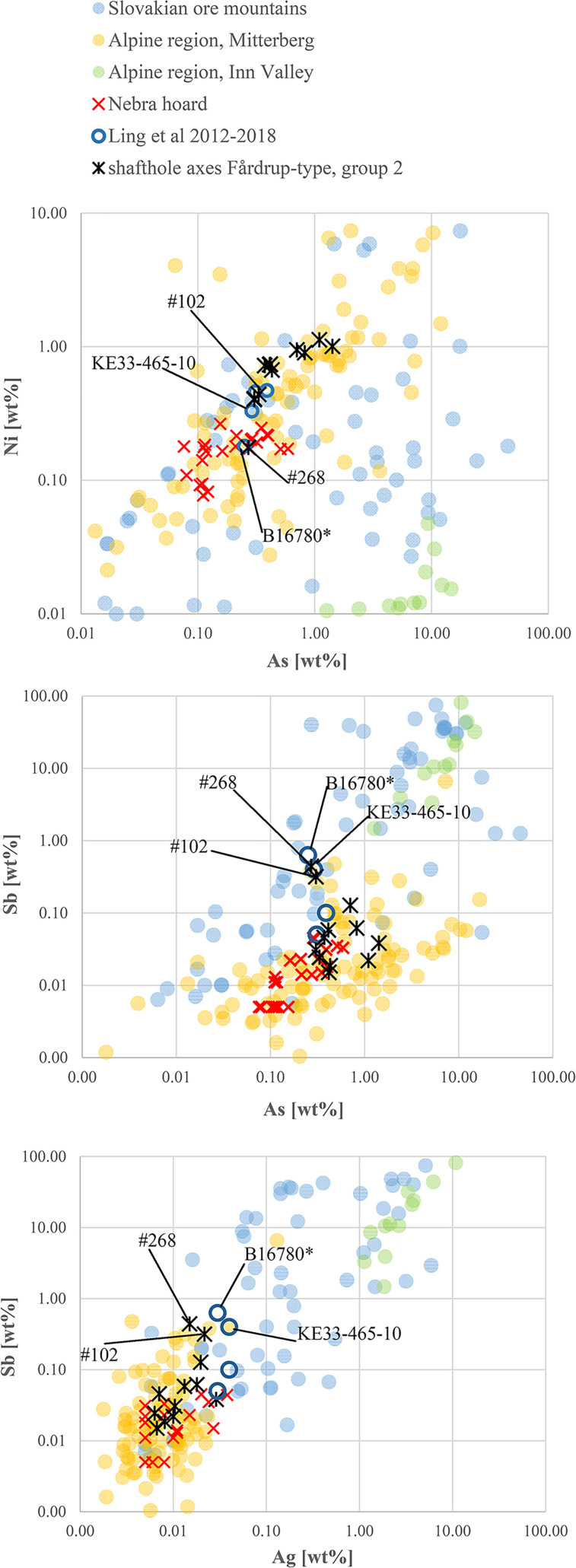
Diagrams of Ni/As, Sb/As and As/Ag concentrations of *isotope group 2* axes. The similarity to the Nebra hoard (data are taken from [40]) is distinct, and the Sb/Ag diagram in particular allows the exclusion of the Slovakian Ore Mountains as a possible source for the Fårdrup-type axes. Valsømagle-type axes (#102, #268), as well as the axe from Löt, Sweden (KE33–465–10), show values that rule out such a clear statement. The comparative data on shaft-hole axes from Sweden, like the double analysis from B16780, derive from the Laboratory of Isotope Geology, Stockholm [6,8]. The ore data are from: Mitterberg ore district [35], Hron Valley, Slovakia [27,28], Inn Valley and Buchberg, Alpine region [29,30]. The analytical uncertainties are comparable with the size of the symbols.

In ***step three***, selected trace elemental patterns were scrutinised once again to eliminate ambiguities in the lead isotope data, specifically regarding the diverse second isotopic group of shaft-hole axes (see Fig 9). The first task was to distinguish between East Alpine and Slovakian provenance regarding the copper of isotope group 2 shaft-hole axes. The Slovakian copper deposits investigated so far delivered high-impurity fahlore copper, but also, faintly discernible in LN II [1], low-impurity copper. The eastern Alpine deposits around Mitterberg are known for their low-impurity copper [35], a feature typical of ores consisting mainly of chalcopyrite. Thus, it seemed obvious to try to distinguish between these sources by trace element comparison. In addition, contemporary artefact assemblages, such as the Nebra hoard, that could be assigned to the ores of Mitterberg on other occasions [40], are used for direct comparison. Fig 10 highlights the similarity between isotope group 2 shaft-hole axes, the Nebra hoard, and the Mitterberg values, particularly in the Ag/Sb diagram. According to the current state of research [27,28], the Slovakian Ore Mountains can mainly be excluded as the supplier of the copper used to produce Nordic shaft-hole axes. Two Valsømagle-type axes (#102, #268), however, differ slightly from the general picture and could derive from a different mine or depth at Mitterberg [97] or alternatively from a different source such as the Slovakian Ore Mountains.

Ling and colleagues analysed two Valsømagle-type axes and two Fårdrup-type axes [3,6,8]. These axes show a clear agreement with the results presented here in trace element and LIA composition (see Figs 9 and 10) regarding both the mainstream results and the uniqueness of Valsømagle-type axes. With one exception [8], all of these were previously identified as of south-west European or Mediterranean provenance [3,6]. In conclusion, isotope group 2 shaft-hole axes of Fårdrup type were likely made from East Alpine copper, more precisely from the Mitterberg mining area.

**Fig 11:**
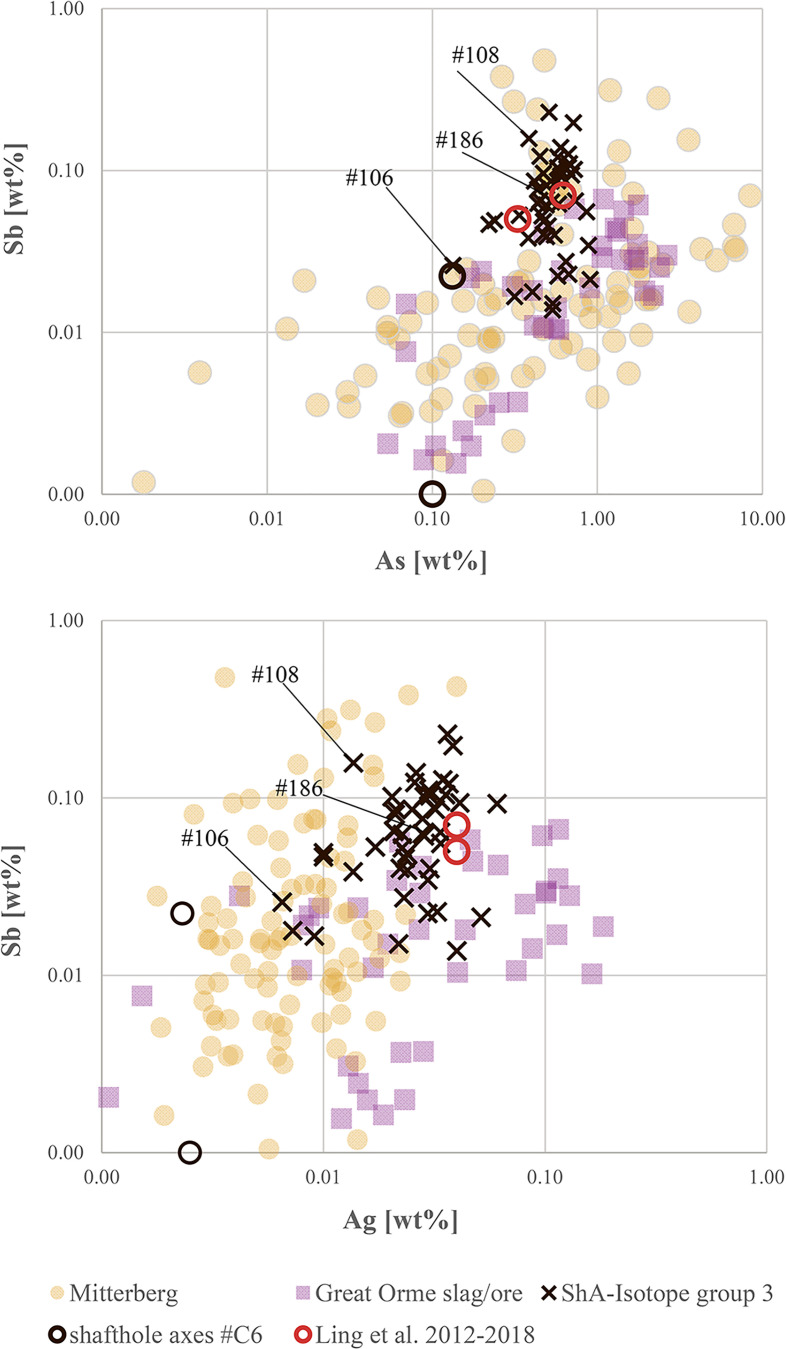
Diagrams of Sb/As and Sb/Ag concentrations of isotope group 3 axes identified through lead isotope data, compared to values from the Mitterberg mining region [35] and Great Orme, Wales [36]. Highlighted is shaft-hole axe #108, which demonstrates the difficulties in allocating the copper of group 3 to one specific ore deposit and the axe from Brørup (#186) and another axe (#106) that most likely derive from Welsh metal. The analytical uncertainties are comparable with the size of the symbols.

In ***step four***, in continuation of the trace elemental strategy, isotope group 3 with the majority of the shaft-hole axes, became the focus of investigation, especially regarding the observed concentration of overall specific lead isotope ratios that at first sight exclude Slovakian and Alpine sources and indicate English or Welsh copper provenance (see Fig 9). The trace element evaluation, however, indicates a close relationship to the East Alpine copper deposits at Mitterberg. Remarkably, the range of group 3 axe values is tightly constrained, covering only a small region of the known values for the Mitterberg and Great Orme deposits (Fig 11). This may well imply that both English/Welsh and East Alpine sources have to be considered as sources for our group 3, especially given that new trace element data from the Great Orme area [36,87] shows that the correlation Ni = As is not unique to copper from Mitterberg. Detailed investigations suggest that mixing of two, if not three, different raw materials (or metallic artefacts) was in fact used to produce the Nordic shaft-hole axes collected in isotope group 3. In conclusion, mixing is capable of accounting for the complexity observed, as elaborated below.

*Firstly*, continuing mixing of British/Welsh metal with East Alpine metal would lead to a homogenisation of the trace element contents and the lead isotope ratios, and thus result in a fairly homogeneous geochemical fingerprint–leading, in other words, to concentration of a large number of artefacts around the same ratios (see Fig 9). A few axes could be allocated directly to English/Welsh ores based on their isotopic and chemical fingerprint: three axes from Bækbølling (#182), Lavesgård (#167) and Højme (#162) fully match the Alderley Edge mine in England [39], which was probably in use at least until the first half of 1600 BC [98–101]. The axe from Brørup (#186) and another axe (#106) closely match the signatures from Great Orme in Wales, where nickel-arsenic metal was extracted specifically during the Middle Bronze Age [36,37,102]. *Secondly*, the majority of group 3 shaft-hole axes show higher Sb–Ag concentrations (Fig 11) than can be recognised in the Great Orme ores, and significantly higher values than measured in the slag prills from Pentrwyn, Llandudno [36,37]. Although more detailed knowledge is still required of the trace element composition of relevant British sources, it can be stated that the many shaft-hole axes of group 3 were probably cast from mixed metal following a trend already identified in NBA IA and LN II [1,2]. This new insight could only have been gained through the evaluation combined above of stylistic features (local production), chemical composition, and isotope ratios. The mixing is well illustrated in Nordic shaft-hole axe #108, which isotopically would be identified as Welsh, while chemically mirroring the metal mined at Mitterberg (Fig 11).

To sum up: The typological and geochemical uniformity of Fårdrup-type shaft-hole axes turned out to hide complexity in provenance patterns. Group three, the largest with 49 axes, revealed local mixing of copper from British ores and from Mitterberg in Austria. Two shaft-hole axes analysed elsewhere [6,8] show very similar values. One axe from Denmark was connected to British ores by Melheim and colleagues [8]. The other Fårdrup-type axe, however, from Ny in Sweden (AM786), had previously been seen as evidence for Cypriot metal in Scandinavia [6]. It should now rather be integrated in this group of axes, with signs of mixed East Alpine and British ore or artefact metal. Mitterberg provenance could also explain isotope group 2 with fourteen axes, while group 1 with only four, showed a clearly deviating pattern compatible with Italian AATV copper. The fact that Valsømagle-type axes, in total seven, were present in all three groups raises the question of possible chronological differences between these two artefact types, aside from their obvious belonging to dissimilar craft traditions.

Could chronology, then, have co-determined the three isotope groups against the backdrop of the clear bipartite division between shaft-hole axe types? In the absence of new absolute dates, establishing a fine-meshed chronology for the two shaft-hole axe forms is problematic; but the compositional patterns described above, interpreted in conjunction with archaeological methods, can provide some indications. Remarkably, Valsømagle-type shaft-hole axes occur in all three groups, which may indicate parallel tracks. Isotope group 1 is particularly noteworthy for its clear Italian AATV isotopic signature, and additionally because inspection of the metal-analytical pre-1600 BC data of objects shows no Italian metal in southern Scandinavia this early [1,8]. The axes of group 1 might then emanate from AATV arrivals as early as late NBA IB, or they could be later, continuing into early NBA II after 1500 BC. Isotope group 1 of AATV provenance comprises two Fårdrup-type axes (#160, #168, Supplement1) and one Valsømagle-type axe (#269). It also includes a Carpathian-style disc-butted shaft-hole axe (of B type, #270), stylistically dated to NBA II around 1400 BC [103], which substantiates the late chronological position of this isotope group.

In conclusion, both Valsømagle-type shaft-hole axes and Fårdrup-type shaft-hole axes occur spread across all three metal groups, eluding definite chronological conclusions [20,95,104]. Nevertheless, even if it is distinctly later than Fårdryp-type metalwork, one might have expected Italian Alpine AATV copper to have been prominent among the Valsømagle items. According to current knowledge, this is not the case. This discussion, however, raises the question of when Italian copper first arrived in the NBA zone, and in what form. Research has so far not been able to identify copper of northern Italian (AATV) provenance in Scandinavia prior to NBA II.

#### 4.4.2 On the track of north Italian AATV metal 1600–1300 BC

Artioli and colleagues [34,75] have recently pointed out the clear importance of the copper deposits in the Italian Alps for the international Bronze Age trade. The arrival of copper to Scandinavia from the AATV region has so far been dated to NBA II [7,50]. When did the AATV copper begin to arrive more regularly, and in what proportions relative to other coppers arriving in Scandinavia? The much larger dataset now available on artefacts and on the major mining areas will help to provide answers to these questions. Several other copper sources than AATV could well have supplied long-range trading enterprises prior to and during NBA II. Recent studies notably identify Austrian, Slovakian and British copper sources 1600–1300 BC [7,8,50].

Long-distance transport of metal to southern Scandinavia is well attested during the first third of the second millennium BC both from the British Isles and from a select few mining sites in central Europe, namely the Inn Valley in the eastern Alps and the Slovakian Ore Mountains [1,6,8]. Although copper deposits in Germany are common, evidence is lacking that they were known and exploited in the Bronze Age: when the provenance of the metals used for the Nebra hoard was investigated, it was possible to exclude most of these copper occurrences as possible sources [25,105]. For the Alpine region more generally, the situation is optimal, because the many adjacent Bronze Age slag sites [73,106–108] reveal, for example, that the technology of copper production in the Italian AATV and the Austrian Alps was quite similar [74,109–117]. Besides, the large volume of lead isotope data from these two Alpine regions [29,30,32,34,73–75] allows for distinctions to be made between them despite their similarity in trace element composition. A well-illustrated example of this new potential concerns the metal objects of the burial of a wealthy woman at Ølby on Zealand, dating to 1400–1300 BC in NBA II. Three items were analysed, revealing similar trace element concentrations. However, the significantly different lead isotope ratios resulted in the suggestion that the copper of which the Ølby items were made originated from three different sources [118]. The most promising approach for tracking the arrival and significance of Italian AATV copper in southern Scandinavia is to compare lead isotope ratios with stylistic information.

**Fig 12:**
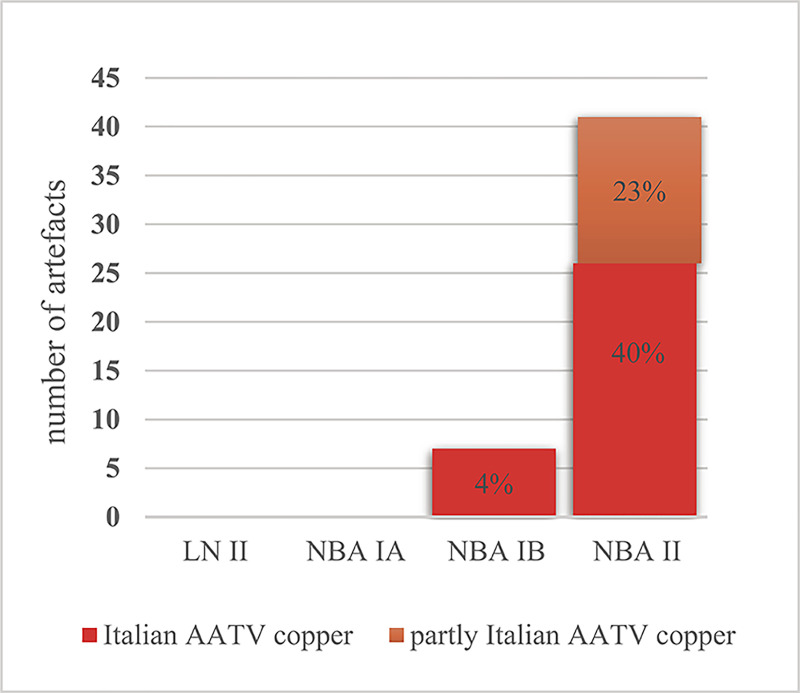
The data evaluation illustrates that Italian AATV copper may have been first used in in southern Scandinavia from NBA IB (1600–1500 BC). Its importance increased drastically towards the mature NBA II c. 1500–1300 BC, and from 1450 BC Italian AATV copper seems to dominate the metal supply. This study shows that probably 40% of the artefacts were made of this copper, while for 23% evidence for mixing metals with other sources is possible.

Isotopically, there is no evidence within the dataset for AATV copper prior to 1600 BC. During NBA IB (1600–1500 BC), only a few artefacts of local Nordic production isotopically match the signatures of the AATV region (Fig 12), namely, the shaft-hole axes #160, #168 and #269, in addition to four high-flanged axes distributed across Denmark (#83, #84, #90, #111). Thus, in NBA IB (1600–1500 BC), Italian ore is represented by seven objects–or four per cent of the analysed material from this period. Seen in the light of the substantial rise of AATV copper in NBA II, that four per cent is remarkable.

At the threshold to the mature NBA II, c. 1500–1450 BC [10,11,104], a rising inflow from AATV copper sources may be indicated by the isotope analysis of eight developed high-flanged axes of Oldendorf type [4], of which two were assigned an AATV provenance. In continuation of the Valsømagle spiral-decorated metalwork, this transitional phase into NBA II is a further step towards the full stylistic conceptualisation of NBA II during which the AATV provenance becomes distinct (Fig 12). This happens in tandem with the Central European Tumulus Culture Br.B-C and indeed through cultural exchanges with the Aegean world [119–121].

#### 4.4.3 The wider panoply of low-impurity chalcopyrite-like copper in NBA IB and NBA II

Fig 4 demonstrates that artefacts dating to NBA IB and NBA II belong to only two to three trace elemental groups (clusters). In NBA IB, 90% is low-impurity copper (C#5/11-C#6), which may be termed sulphidic copper, indicating chalcopyrite-dominated ores. In NBA II, 96% of the analysed artefacts are classified as low-impurity copper. The small remainder group consists of C#2 fahlore. In fact, Nordic use of high-impurity copper (fahlore cluster C#2, C#3, C#7) decreases continuously over time. The full conversion to low-impurity copper around 1600 BC is no doubt significant, albeit the apparent uniformity of trace elemental concentrations hides complexity in provenances, as already demonstrated by the shaft-hole axes.

**Fig 13:**
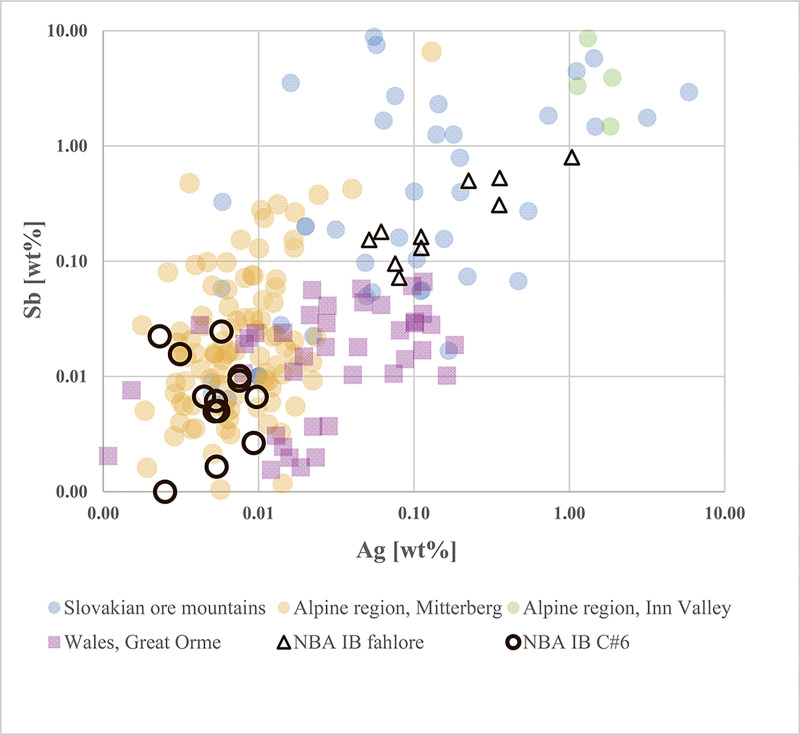
Diagram of Sb/Ag of the NBA IB artefacts consisting of high-impurity copper compared to the low-impurity copper C#6. The Sb/Ag concentration suggests an origin for the fahlore copper in the Slovakian Ore Mountains, while the low-impurity copper is consistent with the tendencies discovered for the shaft-hole axes and point to East Alpine copper. The ore data are from: Mitterberg ore district [35], Hron Valley, Slovakia [27,28], Inn Valley/Buchberg, Alpine region [29,30], Great Orme mining region, Wales [36–39].

**Fig 14:**
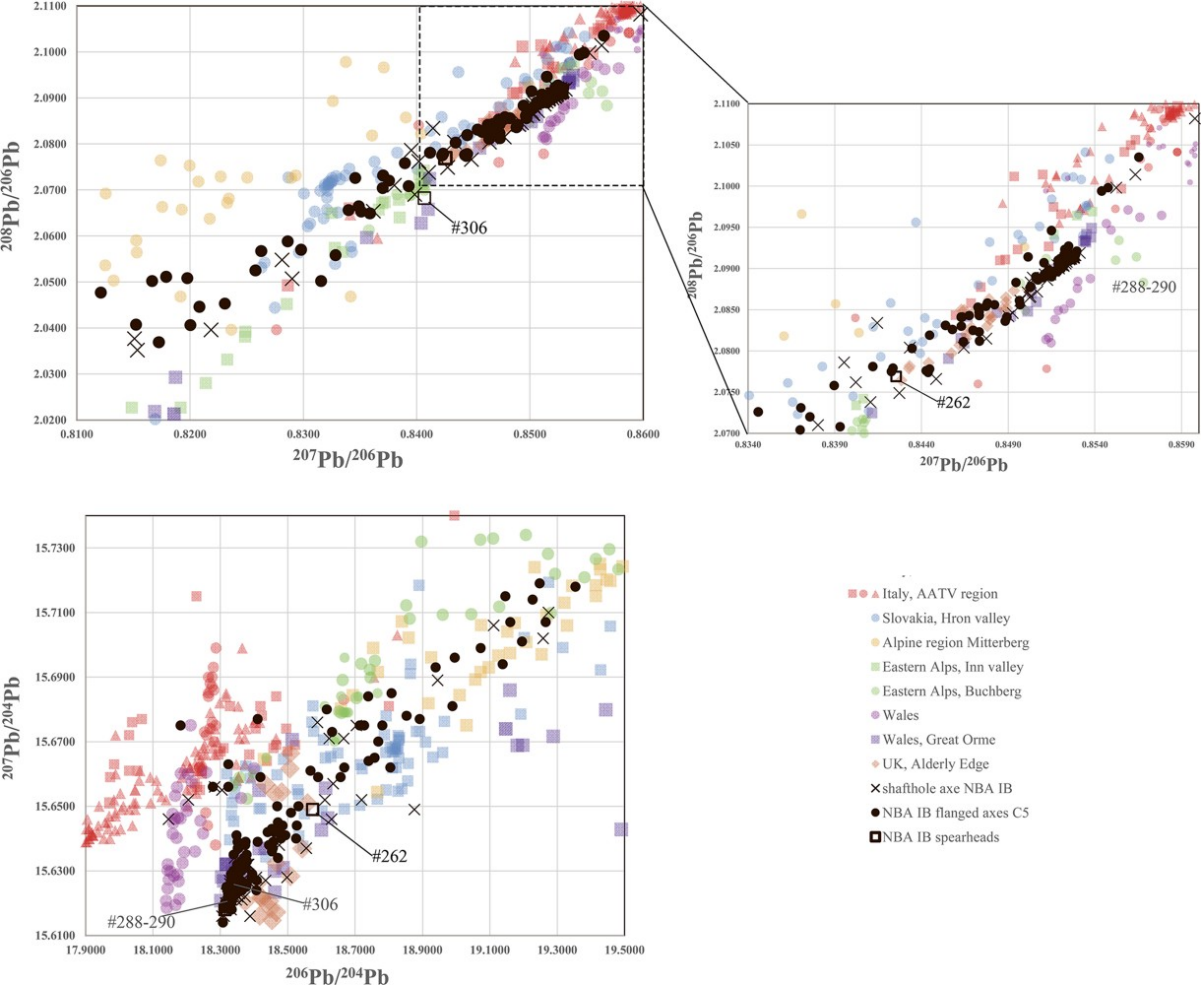
Lead isotope ratios of artefacts consisting of low-impurity copper in NBA IB. The ^206^Pb^/204^Pb/^207^Pb/^204^Pb diagram (bottom) shows that the flanged axes and spearheads are in line with the identified groups of shaft-hole axes. The Valsømagle-type spearhead #262 and the Bagterp-type spearheads #288–290 and #306 (Bagterp and Over-Vindinge) are highlighted. The ore data are from: Mitterberg ore district [35], Hron Valley, Slovakia [27,28], Inn Valley and Buchberg, Alpine region [29,30], Great Orme mining region, Wales [36–39], Alderley Edge mining region, Britain [39,83], central Wales mining region [39,84], Italy AATV mining region [32,73–75]. The analytical uncertainties are comparable with the size of the symbols.

*NBA IB 1600–1500 BC*. Only ten NBA IB artefacts are made of high-impurity copper. Their trace element composition is strikingly similar to the fahlore deposits in the Slovakian Ore Mountains so favoured in LN II and NBA IA [27,28] (Fig 13). These apart, the NBA IB artefacts are made of low-impurity copper. For the extensive group of shaft-hole axes, our tracking of the provenance of their copper to different Alpine and British sources is presented above: in many of these massive axes, we found evidence of mixing copper from British and Mitterberg ore sources. As stated above, only in a few instances was a match found with AATV lead isotope signatures. The 60% of the NBA IB artefacts of the low-impurity copper group that are not discussed above are locally produced high-flanged axes and a few slender weapon-flanged axes, a flanged chisel (classification based on [4]), and five spearheads, including three Bagterp-type spearheads (#288–290) and one Valsømagle-type spearhead (#262) from the eponymous hoards. This new lot displays the same isotopic diversity and can be allocated to the same clearly defined groupings identified above as the shaft-hole axes. Fig 14 reveals more details.

More precisely, in analogy with the shaft-hole axes of *isotope group 2*, 28% of the high-flanged axes analysed, with ^206^Pb/^204^Pb values far above 18.53 and ^207^Pb/^204^Pb values from 15.65, are in line with values from the East Alpine deposits. Further, a group of items made of low-impurity copper probably from the Slovakian Ore Mountains is now distinctly visible in the full dataset for NBA IB; in the large group of shaft-hole axes, this signature was suggested by two Valsømagle-type shaft-hole axes (#102, #268). Thus, by NBA IB, Slovakian low-impurity chalcopyrite copper is distinctly present in the metal stock–having first appeared in very small amounts as early as LN II and having increased markedly during NBA IA [1] (Fig 14). NBA IB even shows a very low presence of “old-fashioned” high-impurity fahlore copper from the same region (Fig 13). The isotopical and elemental overlap within this isotope group 2 (see 4.4.1), however, suggests that mixing of different low-impurity copper sources or artefacts is an option.

Furthermore, a large and dense group consisting of 67 artefacts including high-flanged axes and the spearheads #262, #288–90 and #306 is identified and is comparable to the 47 shaft-hole axes of isotope group 3. The isotopic ratios of these artefacts match the signatures of British and Welsh ore deposits (Fig 14). Even though the flanged axes generally show higher silver values and therefore present Sb–Ag ratios more likely compatible with Great Orme [36,87], the majority of these artefacts escape further classification because their trace elemental values lie in between the known trace element composition of East Alpine and Welsh deposits. By analogy with the shaft-hole axes of isotope group 3 and with cases of intentional reuse of LN II–NBA IA artefacts [see 1,2], this could tentatively be explained by mixing. Alongside the evidence for Great Orme metal, a small assembly of nineteen (15%) artefacts in addition to the three shaft-hole axes identified above indicates a west European origin, but these favour the Alderley Edge mine in Cheshire [39]. The trace element composition of this group does not exhibit any characteristic signature that would allow an allocation to Austrian or Slovakian ores [see 27,28,30,35]. However, state-of-the-art data regarding the trace element concentrations of the English ores around Alderley Edge does not allow any definitive allocation to this source.

In light of this evidence, we propose that despite the decline in identifiable copper clusters, the period from 1600 to 1500 BC (NBA IB) evidences the onset of a much more extended trading network, capable of satisfying the distinctly increased demand for metal in a metalwork production which already reveals early trends in what was to become the classic, emblematic NBA II brand at c. 1450 BC. Alliances with long-standing trading partners in the Slovak region were maintained, but a major new thrust now targeted the East Alpine region, while trading relations with the British Isles intensified compared to LN II–NBA IA levels. Close to 1500 BC, with the first importation of Italian AATV copper, new trading links become visible.

*NBA II Early 1500–1450 BC*. The period around 1500–1450 BC is one of transition to the full-grown NBA II [10,11,89,96]. Already the main ingredients–repetitive mound-building and accomplished metalwork–are in evidence, including spiral symbols in continuation of Valsømagle-type weaponry. Even though only few artefacts dating to the transition period have been sampled, it is significant that they show isotopic diversity in the low-impurity copper (of cluster C#5; C#6). This recalls NBA IB in addition to the classic NBA II, the latter through the presence of Italian AATV copper.

*NBA II Classic 1450–1300 BC*. The full-grown emblematic style–pivoting around an array of spiral patterns and a clear-cut vocabulary of items for men and for women–emerged sometime between 1450 and 1400 BC. The formation process was probably in place around 1450 BC, because the much-cited dendro-dated group of oak coffin burials, 1400–1300 BC, is a geographically and chronologically constrained tradition involving mound-building with so-called iron pans to protect the dead [49,122,123]. The majority of the NBA II artefacts investigated in this study derive from NBA II Classic [12,124], and on this basis can be dated relatively precisely, namely to 1450–1300 BC.

**Fig 15:**
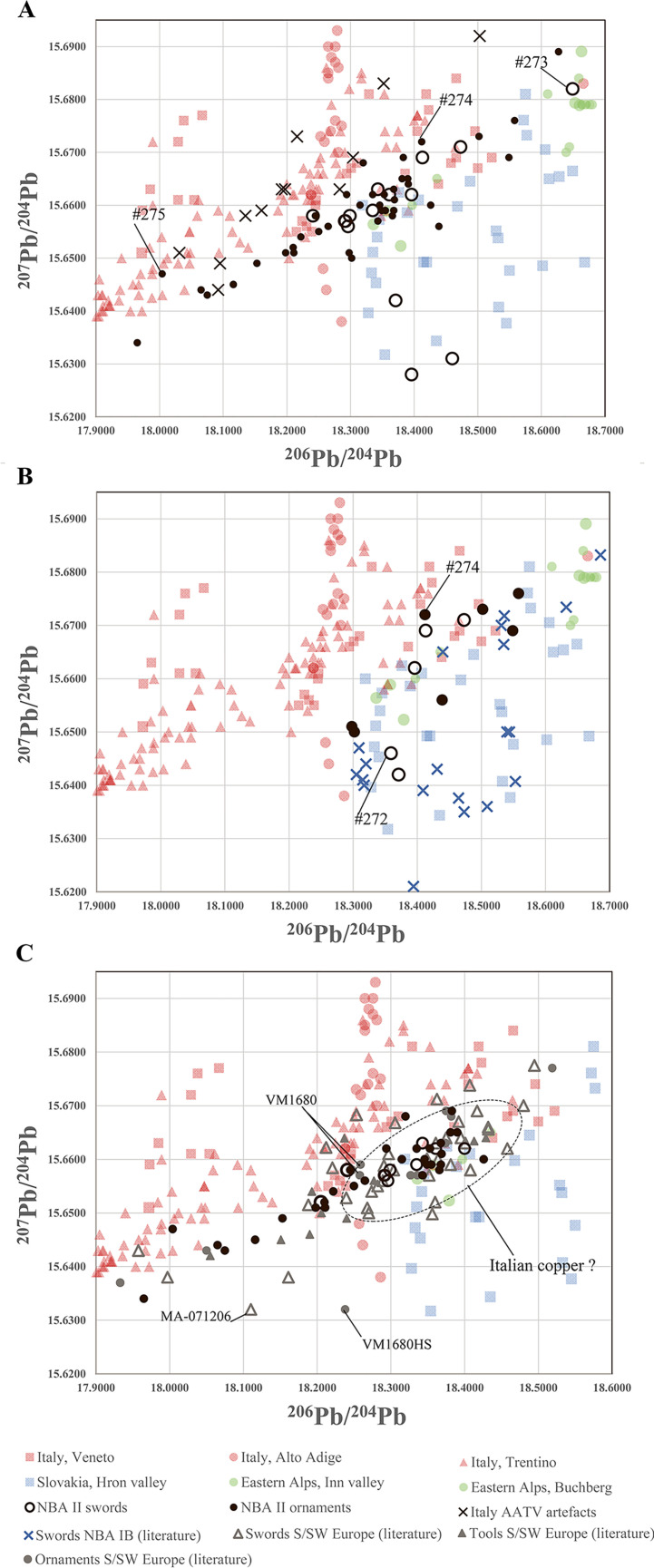
^206^Pb^/204^Pb/^207^Pb/^204^Pb diagram of NBA II artefacts analysed within this study, compared to contemporaneous artefacts. (A) compares the ornaments and swords with the most probable Central and South European ore deposits. Consistent with contemporary artefacts with secured AATV provenance (data taken from [125]), a large group of artefacts is comparable with South Alpine Italian deposits. Artefacts of the Ølby burial, sword #273, neck collar #274 and belt plate #275 are highlighted. (B) compares NBA II artefacts with artefacts of proved Slovakian provenance (data taken from [8,50,51]). The Ølby neck collar #274 and the spearhead from Ullerslev #272 are probably made of Slovakian low-impurity copper. (C) shows that the majority of the artefacts analysed elsewhere (data from [6–8]) fall within the range of the Italian AATV deposits consistent with the data presented here. A significant group (marked) can, at the present state of research, only be defined, but not provenanced. The sheet-tubes (VM1680) from Vognserup Mose [8] and the octagonal-hilted sword MA-071206 [51] are highlighted, plotting an area where several Welsh deposits are present. The ore data are from: Hron Valley, Slovakia [27,28], Inn Valley and Buchberg, Alpine region [29,30], Italy AATV mining region [32,73–75]. The analytical uncertainties are comparable with the size of the symbols.

The lead isotope ratios suggest that 63% of the samples in our suite (57 swords, ornaments and tools) have signatures compatible with the Italian Alpine region. This confirms a trend noted in recent studies [7,8,50]. These samples consist of Italian AATV metal in local metal production in southern Scandinavia (Fig 15A), for example, the Ølby beltplate #275, the two large belt plates from Frankerup #231 and #257, and nine other belt and neck ornaments (#227, #229, #237, #248, #250, #263–264, #266–267, #291). So far, only a single sword blade and disc-butted axe show values similar to the ornaments (#240 and #270). All these artefacts disclose ^206^Pb^/204^Pb values lower than 18.25 and ^207^Pb/^204^Pb values above 15.63, also found in contemporaneous northern Italian artefacts shown to originate in the AATV mining region [125]. This further corroborates the AATV provenance.

A portion of our dataset–eleven artefacts–pinpoints Slovakian low-impurity sulphide deposits: neck collar #274, the belt plates #224–226, #234 and #293, #297, the sword hilt #241 and blade #223, #246 and the spearhead #272. Lead isotope ratios support this provenance (Fig 15B), as does the trace elemental composition, especially the As–Sb ratios. The neck collar from the Jægersborg burial #238 is made from high-impurity fahlore with Slovakian signatures. Over and above the investigation presented here, several swords dating to the classic NBA II have been investigated by Bronze Age scholars in the last years [7,50,51,126], confirming the use of Slovakian low-impurity copper in sword production (Fig 14B). While, surprisingly, only two artefacts in our study–the sword from the Ølby burial #273, and a small belt plate from the Vognserup deposition #294 –can be linked to East Alpine ore deposits at Mitterberg, up to twelve swords from the above-mentioned studies, found predominantly in northern Germany and Denmark, show isotope and trace elemental patterns related to this East Alpine deposit [50,51].

There remain a large number of analyses (23%) with no clear match, either with the Italian ores or with comparable Italian artefacts (Fig 15C). Isotopically, these artefacts can be compared with the values known from Miniera Bedovina in the Trentino [75], the East Alpine Inn valley [29,30] and the Slovakian Ore Mountain ores [27,28]. Definitively stating a provenance for this group, however, is a challenge, given its higher antimony values than known from the East Alpine deposits or the Welsh ores. In the comparative literature [7,8], swords and ornaments with similar isotopic ranges are allocated to Italian AATV deposits. However, once these are compared with the new data presented here (Fig 15C), a significant proportion fall within the isotopically undefined area, including the sheet-tubes from the Vognserup hoard, previously interpreted as made of Iberian copper [8]. This raises questions about the significance of Mediterranean copper in Northern Europe between 1600 and 1300 BC.

The Nordic Bronze Age formed part of a bronze-driven Afro-Eurasian intercommunity from 2100–2000 BC onwards. Baltic amber was reaching Mediterranean urban hubs in significant amounts as early as 1600 BC and continued to flow over the next centuries. Given this, it would not be surprising to find Mediterranean copper in the Nordic metal data, as has been suggested by the Moving Metals project [3,6–8]. Researchers in the present study share ideas regarding the amber-for-metal trade with Ling and colleagues. We are fully in agreement on the extension of the trading network towards southern central Europe in NBA II, a conclusion that rests on corresponding data from Sweden, Denmark, Norway, and northern Germany, all of which pinpoints the presence of Italian AATV copper in NBA II [7,8]. The Moving Metals project claimed to identify Spanish, Portuguese, Sardinian and Cypriot copper in the production of a number of Nordic artefacts, notably some shaft-hole axes as well as high-flanged axes of Underåre-type [6] in NBA IB, 1600–1500 BC. One NBA II sword from Odsherred in Holbæk county analysed in the Moving Metals study revealed high ^208^Pb/^206^Pb and ^207^Pb/^206^Pb lead isotope values, which were interpreted as indicating a possible Sardinian origin of the metal [7]. However, the present study, based on more than 500 samples, has not been able to detect copper of Mediterranean or Iberian provenance in the analysed first 700–800 years of the Bronze Age in Denmark.

If a pool of this metal had been available locally, Mediterranean signatures would have been identifiable, particularly among the female ornaments measured. Unlike swords, the ornaments of the Nordic Bronze Age, such as belt plates and neck collars, are stylistically and technologically restricted to southern Scandinavia [47,49]. Accordingly, they most certainly are local products, and their metal must have been imported, but not the artefact itself. Swords do not always define a local production. The first swords in southern Scandinavia arrived around 1600 BC from south-east Europe. They were traded from the Carpathian basin and Transdanubia as far as Middle Sweden and northernmost Denmark and were often recast locally as lookalikes [20,41,50,127–129]. A similar pattern in sword production is found in the developed Bronze Age: distinct sword types are widely distributed across parts of Europe, and a specific local production is hardly visible [130,131]. Thus, the metal of swords will not necessarily illuminate the metal trading networks behind local metalcraft as clearly as with ornaments.

The group of analyses that elude clear identification even though the range of trace elements and isotope ratios is broadly compatible with central European ores includes several artefacts for which a Mediterranean or Iberian origin has been discussed [6–8]; but at present, in view of the intense local mixing, a more precise identification is unreachable. It seems, though, that west European metal may have played a role in this process (see Fig 15C), as the octagonal-hilted sword (MA-071206, [50]) and the sheet-tube from Vognserup (VM1680HS, [8]) could probably consist of Welsh metal. Considering that British and Welsh copper always played a role in southern Scandinavian metallurgy, one is tempted to explain the mixed signatures of these artefacts in terms of the continuous remelting of the metallic material culture.

To conclude: From 1600 BC, almost all the substantial quantities of metal reaching Scandinavia are to be classified as copper smelted from sulphidic ore, dominated by chalcopyrite. Despite a striking uniformity, scrutiny of the trace element components and isotopic signatures have revealed a diversity contingent on the origin of the metal. In NBA IB 1600–1500 BC, a wide array of provenances are present: the eastern Alps at Mitterberg, Great Orme in Wales, Alderley Edge in central England, and finally the Slovakian Ore Mountains in continuation of an earlier tradition. By comparison, Italian AATV is only just discernible in the data. The transitional phase to classic NBA II is transitional also in terms of its copper imports, and AATV seems to have been on the rise. During classic NBA II, 1450–1300 BC, Italian AATV copper rises markedly to over 60%, followed by the old acquaintance of Slovakia. The data of this study indicates that south-east Alpine AATV copper was the most southerly supplier for Danish metal production from the onset of NBA II–with a tiny beginning in NBA IB.

## 5. Conclusion

The novelty of this study rests on the large number of early metal artefacts investigated from the early phases of the Neolithic through to the established Nordic Bronze Age. A representative coverage is especially relevant to the Bronze Age periods 2100–1300 BC. It has been possible to identify the major sources of metal arriving in each period (S1 Fig), and additionally to track how changing provenances tie up with new artefact styles and most likely with new forms of social organisation. More precisely, the inquiry has shown a tight relationship between early metal use in each major period and changes in the provenance and class of copper arriving in southern Scandinavia. Furthermore, as will be elaborated in the paragraphs that follow, these shifts in metals entailed reorganisation not only in the long-distance routes of transfer, but also, through responses to changed geopolitics at a European level, in the societal setup in the north. The dating of these transitions by the associated archaeological material is not sufficiently precise to demonstrate with certainty whether the changes happened at the same time or sequentially over the period in question. In either case, however, causality is involved. In the light of the analytical results described above, the configuration of several changes is indeed significant.

Before the advent of the Bronze Age c. 2100–2000 BC, material culture in the Neolithic is characterised by on-and-off presence of items of copper and gold. The two Neolithic periods with a faintly discernible rise in metals are the Funnel-Beaker culture of the mid-fourth millennium BC and the Bell-Beaker-affiliated LN I in the later third millennium BC. For both periods, metallurgical experiments likely contributed to increased interest in metal items, with the clearest evidence in the earlier period [4,13–15,52,132]. The present study has provided new insights into the Bygholm type of metalwork made of arsenic-rich but otherwise pure copper: the new analyses have revealed a strong connection with south-eastern Europe. This prevalent Riesebusch-Mondsee copper is likely consistent with copper mines in Serbia and/or Bulgaria. We can therefore point to a link between southern Scandinavia and south-eastern Europe’s rich Chalcolithic, however in all likelihood with the Austrian Mondsee group as mediator of copper to the north. The Bygholm metalwork appears to form part of the new networks that enabled the establishment of a fully Neolithic society in southern Scandinavia. Its appearance coincides remarkably with the appropriation of other novelties, such as jadeite axes, long barrows, causewayed enclosures and, eventually, megalithic monuments of dolmens and passage graves [15].

The Bell-Beaker-related copper differs radically from the uniform Riesebusch-Mondsee copper. It is isotopically and compositionally so diverse that a single provenance seems unlikely. The later Earlier Neolithic and the entire Younger Neolithic with Corded Ware migrations (3500–2300 BC) are often described as periods devoid of metals. However, the analysed dataset hints that isolated prestigious copper axes did find their way north even in the long intermediate periods of the Neolithic. More research is required to probe into this question and the extent to which metallurgical knowledge accompanied the arrival of metal items in the Neolithic [15].

In relation to the clear and continuing increases in copper imports to southern Scandinavia from 2100–2000 BC onwards, Neolithic copper use was insignificant. That fundamental change denotes a transition from predominantly regional transmission of commodities among fairly autonomous Neolithic societies to a fully globalised Bronze Age economy entirely reliant on long-distance trading of copper and tin [18,19]. This dependency transformed societies irreversibly as regular metal supplies became necessary for the political economy to survive intact. In fact, the transition 2100–2000 BC was the point of no return. The evidence of regularly shifting ore sources and relocation of the transfer routes in operation at different points in time may indicate that Bronze Age hyper-connectedness had innate weaknesses, with associated consequences for the local setups of power. Shifts in one part of the system had the potential to impact other, even distant parts.

In the period 2100–1700 BC (LN II), three supply regions are identified (see S1 Fig): the eastern Alps, Slovakia, and the British Isles [1,2,133]. First to arrive, by way of EBA hubs in Únětician central Germany, was high-impurity fahlore copper from the Austrian Inn Valley and from the Slovakian Ore Mountains, in quite large quantities. This was at the height of the Únětice culture, which boasted princely graves as well as enormously rich hoards such as Dieskau 2 at Halle-Saale, which contained Baltic amber in addition to numerous metal objects [134]. Abundant amber in the metal-rich cemeteries of Bohemia (Czech Republic) indicate that Baltic amber arrived there along this eastern route, but apparently it did not go any further [45,135]. At the same time, a western sea route linked southern Scandinavia with the British Isles and provided desired high-tin copper in exchange for Baltic amber (found in clusters in the rich EBA graves of the Wessex region of Wiltshire [17]). Back home, the growing production, consumption and circulation of metals provided comparative advantages in the competition for sociopolitical leadership. These are visible in the building of ancestral gallery graves for collective burial, over-size longhouses, hoarding of metal objects, and metal-led material culture more generally. Most of these manifestations can be considered a compromise between a rooted communal tradition and new leadership forms emerging from the metal enterprise [17,21].

Imports into Scandinavia took place in the form of rings and axes, treated as ingots [1]. Low-Ni fahlore copper mined in the Inn Valley of the eastern Alps was traded intensively, shaped in the characteristic Ösenhalsringe form. The 30% high-tin artefacts in LN II underline the importance of English and Welsh copper in local Nordic metal production. Practically, imported large British bronze flat axes were mixed with hack metal from the Inn Valley and Slovakia: the geochemical traits of British copper deposits identified in objects of local Scandinavian styles, such as the Værslev/Æbelnæs-type axes from Valore #33 and Æbelnæs #39 on Zealand, confirm the remelting of foreign artefacts into products suited to local taste and demand. The extreme high-tin (10–14%) content of the British bronze flat axes was especially sought after [1,2,17], and must have prepared the ground for the full adoption of tin-alloyed bronze from c. 1700 BC. This robust evidence for the implementation of British-Scandinavian trade as early as 2000 BC is fresh and recent.

Beginning c. 1700 BC, NBA IA is a period of metallurgical and military innovation. This is manifest in standard tin-bronze alloys and the numerous socketed spearheads that were now in production. NBA IA is also about crisis, apparent in the abandonment or reorganisation of settlements with traditional two-ailed longhouses [136]. Importation of British artefacts is no longer in evidence, even though British and Welsh metal is a well-used source in local southern Scandinavian metalwork, as shown in the increased tin-values of the elaborated axes. A British signature still transpires in the dataset of metals, which now comprise nearly two-thirds Slovakian fahlore well-known since LN II and one-third of novel low-impurity chalcopyrite-like copper, also from the Slovakian Ore Mountains (see S1 Fig). This mélange, visible in the trace element patterns and the lead isotope ratios [1], may stem in part from the local production practice, seen clearly in LN II, of one-to-one mixing of artefact metal from different sources. Increased recycling would be consistent with changing geopolitics in non-Mediterranean Europe at the threshold to the Middle Bronze Age. A part of the Slovakian fahlore could thus be recycled from LN II, which would explain the British traces. However, the Slovakian chalcopyrite-like low-impurity copper demonstrates that new loads of metal did reach Scandinavia at some point during the seventeenth century BC. By now, tin is standard, but its provenance is unknown: it could be either from Cornwall or from the Erzgebirge mountain range.

Many societies changed culturally and socially in the seventeenth century BC, but given current knowledge, it is hard to pin precise social labels and timelines on this change in northern, western and central Europe. This is the period of the demise of the formerly prominent Únětice culture, which disappears from the record c. 1600 BC [46]. The metal-for-amber trade must have collapsed as the reliable economic basis for the contributing persons or groups [137–139]. But by the same token, with the tight control of this trade formerly exercised by the Únětician bottleneck dwindling, the path opened towards the resource-rich crossroads in the Carpathian basin, for the Scandinavians as for others: the amber-for-metal trading network, begun in LN II, extended its range in NBA IA towards the south-east [45]. The plentiful Baltic amber in the Mad’arovce-Věteřov cemeteries of south-west Slovakia at the transition from EBA to MBA [45,140] verifies that the eastern route did not disappear. It may well have been destabilised, but it was extended nonetheless. Much of the metal reaching southern Scandinavia at this time went into spearheads. The lengthening of the eastern route accords well with the origin of the spearhead idea in the Carpathian basin (ultimately the Steppe Zone) and with the first arrival of chalcopyrite-like copper in Scandinavia from Slovakia. These transactions likely interwove with new social ideas, stimulating local metal production to keep pace with innovations. The demise and the final collapse of the Únětice culture may have brought with it some shortage of metals in the north, possibly accounting for the suggested increase in the reuse of existing metals. Another indicator in support of such a scenario is the new developments in the succeeding period, when the Carpathian connection becomes even more distinct.

By NBA IB, the visible boom in copper imports to Scandinavia is coeval with near-industrial mining in major ore fields, which took off in the sixteenth century [35,41,141,142]. The dataset further points to a much-extended trading network (see S1 Fig). The more than two hundred artefacts analysed from this first breakthrough of the NBA strongly indicate the reorganisation of the trade towards routinisation and commodification, likely with separate ingots for copper and tin. This is the conclusion that emerges from the dataset, showing British metal once again playing a central role in Scandinavia. Albeit there are no metal objects imported from the British Isles, there is clear evidence of much-increased use of English (Alderley Edge) and especially Welsh (Great Orme) copper. Clearly, the sea route between southern Scandinavia and the British Isles had been re-established, now probably carrying ingots. The overall boom including the British revival may ultimately trace back to the Únětice collapse, which opened new opportunities with new trading partners (see S1 Fig). Besides, much copper kept arriving, not only from maintaining well-established contacts in Slovakia, but now also from Mitterberg in the eastern Alps (Fig 14). Despite the three main provenances visible in isotopic and trace-component data, almost all of this copper is now of low-impurity quality. In the light of the previous wide panoply of coppers, this uniformity is remarkable. This low-impurity As = Ni “chalcopyrite” copper must have had qualities much in demand among many communities. It fits the picture of growing standardisation of the metals enterprise across the board, from production to trading.

The direct Scandinavian access to the Carpathian basin and the Transdanubian plains that was opened up by the Únětice collapse c. 1600 BC [46] increased in importance in the sixteenth century BC as signs of contact appear with tell settlements in the Koszider period (≈Fårdrup-Hajdúsámson-Sögel metalwork) and the earliest Middle Danube tumulus groups in Bronze Age B1 (≈Valsømagle metalwork). What we see here may be the consolidation of the eastern route, the extension of which now went as far as the Aegean, where Baltic amber was included in the extraordinary shaft grave assemblages of Mycenae [143–146]. Halfway between southern Scandinavia and the Carpathian–Transdanubian crossroads, the Nebra hoard, with its twin sets of Hajdúsámson-derived weaponry made of Mitterberg copper and perhaps even tin from Cornwall [40,41], marks the place where the eastern route branched in two. One of these branches was a westerly itinerary along the River Elbe to the Sögel-Wohlde region of north-west Europe, while a north-easterly and similarly riverine itinerary headed towards the Baltic Sea, crossing over to the Danish isles and Scania. In Scandinavia, these changes concurred with formation of a new social order, identified by an emblematic style among prominent warriors in the incipient NBA. This period saw the first rush of construction of large burial mounds [122,123], erected as monuments to commemorate individual founders of this warrior class who carried Valsømagle-type gear. Crucially in NBA IB, Italian AATV copper is faintly discernible among the copper provenances in the Bronze Age dataset. This development signals the onset of changes that came to full fruition after c. 1500–1450 BC.

In NBA II (1500–1300 BC), British copper is no longer detectable in the dataset. This may mean that the sea-based trade between southern Scandinavia and the British Isles had declined or ceased and that Italian AATV copper had seized dominance (see S1 Fig), which is in accordance with established knowledge [7,8]. This takeover coincided with establishment of the full-grown NBA, with burial mounds by the thousand and a unifying metalwork style that branded the upper social echelon of men and women in distinct yet shared ways. This tie-up with a prevailingly western riverine and land-based route now connected the NBA region with the South German Tumulus culture and the first transalpine amber traffic. Abundant finds of Baltic amber in the rich mound burials of the South German Tumulus region [147] indicate a major shift in alliances and trading routes. Material culture wise, there are several similarities between the classic NBA II and the South German Tumulus group e.g. [5,50]. Baltic amber was now travelling across the Alps and likely passed through Val Camonica to the Po valley, continuing from there to Apulian coastal entrepots like Roca Vecchia [119,148,149] in South Italy.

The three-track approach adopted in this study allows us to draw on the trace elemental and isotopic data diagnostically in conjunction with our detailed classificatory knowledge [based on 4,47,50,52,118,150–171] of the sampled artefacts. None of these methods alone would be capable of revealing the provenance of the metals used for crafting the first sizeable quantity of objects at the turn of the Neolithic to Bronze Age, and further into the first three hundred years of the remarkable Nordic Bronze Age. The study is exploratory, but its structured framework incorporating time, geography and technology, past and present, thus deploying advances both in archaeology and in science has been extremely productive. The inquiry has fulfilled its primary aim: namely, to unveil these ore sources and trading networks that enabled the rise and establishment of the Nordic Bronze Age. Importantly, it has demonstrated that the Nordic Bronze Age was founded on metals from shifting ore sources, in tight correlation with altered trading routes and altered geopolitical and societal setups. The sequence of distinct sociocultural transformations from 2100 to 2000 BC (the turn of LN I to LN II), 1600 BC (the turn of NBA IA to NBA IB) and 1500 BC (the turn to NBA II) has been found to correlate with significant changes in the provenance and class of copper arriving in southern Scandinavia.

## Supporting information

S1 Fig Simplified map of the shifting copper trade routes to southern Scandinavia from 2000 BC -1300 BC. The animated map includes the most relevant copper and tin deposits (like the Slovakian Ore Mountains (Slovakia), The Mitterberg area and the Inn Valley (eastern Alps), the AATV mining region (northern Italy), the Central Wales mining region and the Great Orme mine (Wales), the Alderley Edge mine (England) and the major cultural groups discussed in the article. Map images are provided by Natural Earth (public domain) under a CC BY 4.0 license, designed by H. W. Nørgaard using the software Adobe Photoshop 2020/ Microsoft PowerPoint.
(GIF)

S1 Table Complete repository information of the artefacts presented within this study [4, 47, 50, 52, 118, 150–171]. (PDF)

S2 Table Datasheet (Excel) of the lead isotope (MC-ICP-MS) and trace element (EDXRF) analyses executed on 311 artefacts dating from 3800 BC to 1300 BC. The ^14^C ranges are based on a combination of radiocarbon and dendrochronological determinations [9–12].
(XLSX)
