# Supplementary figures and images for: Shifting networks and mixing metals: Changing metal trade routes to Scandinavia correlate with Neolithic and Bronze Age transformations
Source: PLoS One. 2021 Jun 16;16(6):e0252376. doi: 10.1371/journal.pone.0252376 (PMC8208583; doi:10.1371/journal.pone.0252376)

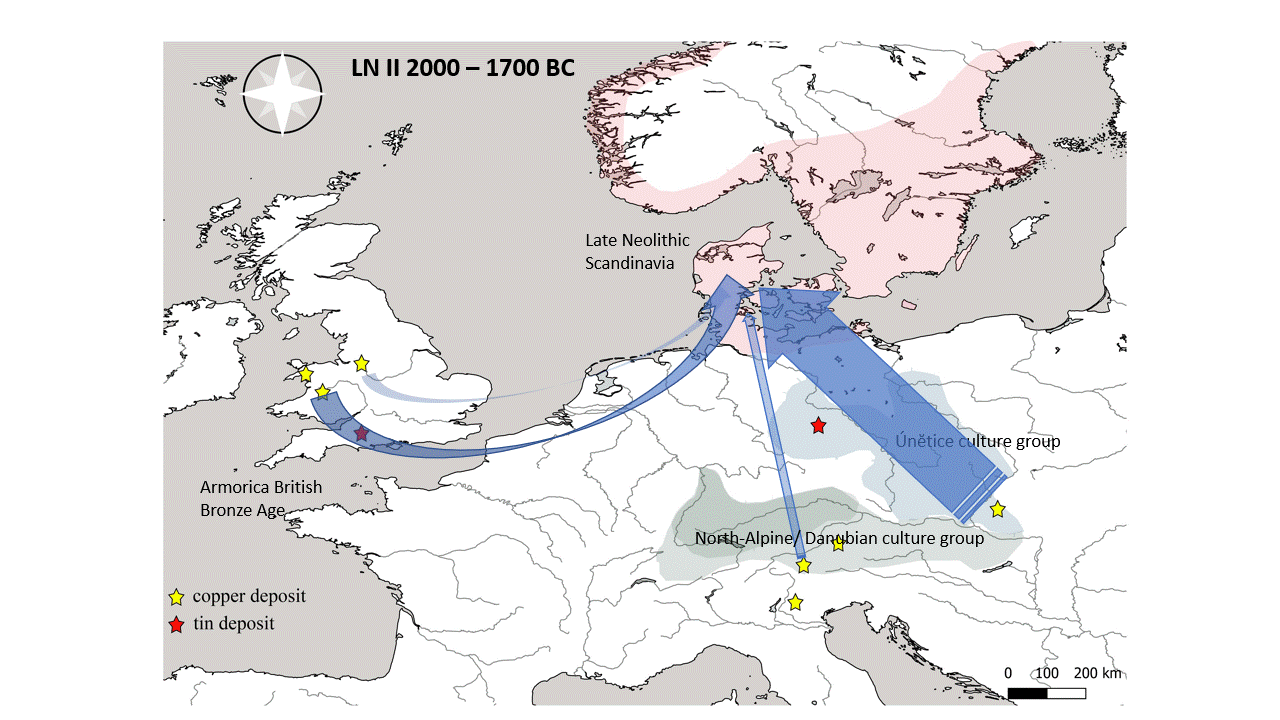

Supplement: S1 Fig — The animated map includes the most relevant copper and tin deposits (like the Slovakian Ore Mountains (Slovakia), The Mitterberg area and the Inn Valley (eastern Alps), the AATV mining region (northern Italy), the Central Wales mining region and the Great Orme mine (Wales), the Alderley Edge mine (England) and the major cultural groups discussed in the article. Map images are provided by Natural Earth (public domain) under a CC BY 4.0 license, designed by H. W. Nørgaard using the software Adobe Photoshop 2020/ Microsoft PowerPoint. (GIF) [file pone.0252376.s001.gif]
