# Supplementary material for: Shifting networks and mixing metals: Changing metal trade routes to Scandinavia correlate with Neolithic and Bronze Age transformations
Source: PLoS One. 2021 Jun 16;16(6):e0252376. doi: 10.1371/journal.pone.0252376 (PMC8208583; doi:10.1371/journal.pone.0252376)
Supplement: S1 Table — (PDF) [file pone.0252376.s002.pdf]

S1 Table: Complete repository information of the artefacts presented within this study [4, 47, 50, 52, 118, 150-171].

findplace	ID	object/ artefact	lab-no.	collection-no.	museum/ collection	date/ period	context references
Albøge, Djurs Sønder, Randers	**#212**	flanged axe, type Oldendorf	MA-171428	FHM 5264	Forhistorisk Museum Moesgaard	NBA IB	Vandkilde 1996
Aldrup Kjær, Hovlbjer, Viborg	**#86**	flanged axe, type Oldendorf	MA-171173	B12365	National Museum Copenhagen	NBA IB	Aner and Kersten, 2008; Vandkilde 1996
Allerup, Sallinge, Svendborg	**#160**	shafthole axe, type Fårdrup	MA-171376	Mi B1264	Fyns Stiftmuseum	NBA IB	Vandkilde 1996; Aner and Kersten, 1977
Allese, Lunde, Odense	**#147**	flanged axe, type MaegerkingenV	MA-171363	A1779	Fyns Stiftmuseum	NBA IB	Vandkilde 1996; Aner and Kersten, 1977
Allingåbro, Sønderhald, Randers	**#196**	shafthole axe, type Fårdrup	MA-171412	VHM 21329	Vendsyssel Historiske Museum	NBA IB	Vandkilde 1996; Forssander 1936
Avnslev Overby, Vindinge, Svendborg	**#82**	flanged axe, type Oldendorf	MA-171169	B11921	National Museum Copenhagen	NBA IB	Vandkilde 1996; Aner and Kersten, 1977
Bagterp, Vennebjerg, Hjørring	**#126**	flanged axe, type Underaare	MA-171342	B9036	National Museum Copenhagen	NBA IB	Vandkilde 1996
Bagterp, Vennebjerg, Hjørring	**#127**	flanged axe, type Underaare	MA-171343	B9037	National Museum Copenhagen	NBA IB	Vandkilde 1996
Bagterp, Vennebjerg, Hjørring	**#128**	flanged axe, type Oldendorf	MA-171344	B9038	National Museum Copenhagen	NBA IB	Vandkilde 1996
Bagterp, Vennebjerg, Hjørring	**#129**	flanged axe, type Underaare	MA-171345	B9039	National Museum Copenhagen	NBA IB	Vandkilde 1996
Bagterp, Vennebjerg, Hjørring	**#288**	spearhead, Bagterp type	MA-180964	B9045	National Museum Copenhagen	NBA IB	Vandkilde 1996
Bagterp, Vennebjerg, Hjørring	**#289**	spearhead, Bagterp type	MA-180965	B9041	National Museum Copenhagen	NBA IB	Vandkilde 1996
Bagterp, Vennebjerg, Hjørring	**#290**	spearhead, Bagterp type	MA-180966	B9044	National Museum Copenhagen	NBA IB	Vandkilde 1996
Bastrup, Lynge, Frederiksborg	**#58**	flanged axe, type Oldendorf	MA-171145	NM 26106	National Museum Copenhagen	NBA IB	Vandkilde 1996, Aner and Kersten, 1973
Beddinge, Vermenhögs, Scania	**#310**	flanged axe, Anglo-Irish type	MA-181036	ML6574	Lund University Historical Museum	LN II	Vandkilde 1996; Forssander 1936
Billesbølle, Vends, Odense	**#80**	flanged axe, type Oldendorf	MA-171167	NM 10723	National Museum Copenhagen	NBA IB	Vandkilde 1996; Aner and Kersten, 1977
Birket, Lollands Nørre, Maribo	**#65**	flanged axe, type Oldendorf	MA-171152	B9507	National Museum Copenhagen	NBA IB	Vandkilde 1996; Aner and Kersten, 1977
Bjælkerup, Stevns, Præstø	**#2**	flanged axe, type Emmen	MA-166620	NM B7209	National Museum Copenhagen	LN II	Vandkilde, 1996; Aner and Kersten, 1976
Bolderslev, Rise, Åbenrå	**#17**	shafthole axe, type Fårdrup (ud)	MA-166668	NM B13968	National Museum Copenhagen	NBA IB	Aner and Kersten, 1981
Brahetrolleborg, Sallinge Svendborg	**#159**	shafthole axe, type Fårdrup	MA-171375	1.5.1886	Fyns Stiftmuseum	NBA IB	Vandkilde 1996; Aner and Kersten, 1976
Brattingsborg, Samsø, Holbæk	**#25**	shafthole axe, type Fårdrup (ud)	MA-166676	NM B9612	National Museum Copenhagen	NBA IB	Aner and Kersten, 1976
Bregninge, Sunds, Svendborg	**#138**	shafthole axe, type Fårdrup	MA-171354	14909	Svendborg Museum	NBA IB	Vandkilde 1996; Aner and Kersten, 1977
Bringe, Værlose, København	**#237**	neck collar	MA-173719	NM B3486	National Museum Copenhagen	NBA II	Nørgaard 2018
Broby, Alsted, Sorø	**#70**	flanged axe, type Oldendorf	MA-171157	B5017	National Museum Copenhagen	NBA IB	Vandkilde 1996; Aner and Kersten, 1976
Broholm, Gudme, Svendborg	**#158**	flanged axe	MA-171374	A1883	Fyns Stiftmuseum	NBA IB	Vandkilde 1996; Aner and Kersten, 1977
Brokbjerg Mose, Hellum, Ålborg	**#4**	flanged axe, type Emmen	MA-166631	NM B198	National Museum Copenhagen	LN II	Vandkilde, 1996
Brunde, Rise, Åbenrå	**#30**	shafthole axe, type Fårdrup (ud)	MA-166681	NM B377	National Museum Copenhagen	NBA IB	Aner and Kersten, 1981
Brørup, Malt, Ribe	**#186**	shafthole axe, type Fårdrup	MA-171402	KHM 8354	Kulturhistorisk Museum Randers	NBA IB	Aner and Kersten, 1986
Bygholm, Nim, Skanderborg	**#12**	flanged axe, type Oldendorf	MA-166663	NM 10187	National Museum Copenhagen	NBA IB	Aner and Kersten, 2014; Vandkilde, 1996
Bækbølling, Malt, Ribe	**#100**	shafthole axe, type Fårdrup	MA-171187	B7548	National Museum Copenhagen	NBA IB	Vandkilde 1996; Aner and Kersten, 1986
Bækbølling, Malt, Ribe	**#181**	shafthole axe, type Fårdrup	MA-171397	O-3404a	Museet på Koldinghus	NBA IB	Vandkilde 1996; Aner and Kersten, 1986
Bækbølling, Malt, Ribe	**#182**	shafthole axe, type Fårdrup	MA-171398	O-3404b	Museet på Koldinghus	NBA IB	Aner and Kersten, 1986
Bøgholt, Vennebjer, Hjørring	**#85**	flanged axe	MA-171172	B2014	National Museum Copenhagen	NBA IB	Vandkilde 1996
Cathrinedal, Tudse	**#305**	flanged axe, type C1	MA-181038	NM 14331	National Museum Copenhagen	NBA IB	Vandkilde 1996
Christiansminde, Horns, Frederiksborg	**#52**	flanged axe, type Underåre	MA-171139	B3621	National Museum Copenhagen	NBA IB	Vandkilde 1996, Aner and Kersten, 1973
Christiansminde, Horns, Frederiksborg	**#83**	flanged axe, type Oldendorf	MA-171170	B14365	National Museum Copenhagen	NBA IB	Vandkilde 1996, Aner and Kersten, 1973
Christiansminde, Horns, Frederiksborg	**#84**	flanged axe, type Oldendorf	MA-171171	B14366	National Museum Copenhagen	NBA IB	Vandkilde 1996, Aner and Kersten, 1973
Darum, Gørding, Ribe	**#175**	shafthole axe, type Fårdrup	MA-171391	RIB 7656	Sydvestjyske Museer	NBA IB	Aner and Kersten, 1986
Denmark	**#41**	flanged axe, type Gallemose	MA-171079	NM	National Museum Copenhagen	LN II	Vandkilde 1996
Denmark	**#55**	flanged axe, type Oldendorf	MA-171142	B8679	National Museum Copenhagen	NBA IB	Vandkilde 1996
Denmark	**#56**	flanged axe, type Oldendorf	MA-171143	NM 26076	National Museum Copenhagen	NBA IB	Vandkilde 1996
Denmark	**#101**	shafthole axe, type Fårdrup	MA-171188	NM	National Museum Copenhagen	NBA IB	Vandkilde 1996, Aner and Kersten, 1973

S1 Table: Complete repository information of the artefacts presented within this study [4, 47, 50, 52, 118, 150-171].

findplace	ID	object/ artefact	lab-no.	collection-no.	museum/ collection	date/ period	context references
Denmark	**#102**	shafthole axe, type Valsømagle	MA-171189	B13538	National Museum Copenhagen	NBA IB	Vandkilde 1996, Aner and Kersten, 1973
Denmark	**#103**	shafthole axe, type Fårdrup	MA-171190	B13537	National Museum Copenhagen	NBA IB	Vandkilde 1996, Aner and Kersten, 1973
Denmark	**#104**	shafthole axe, type Fårdrup	MA-171191	NM 26014	National Museum Copenhagen	NBA IB	Vandkilde 1996, Aner and Kersten, 1973
Denmark	**#105**	shafthole axe, type Fårdrup	MA-171192	NM 9525	National Museum Copenhagen	NBA IB	Vandkilde 1996, Aner and Kersten, 1973
Denmark	**#106**	shafthole axe, type Fårdrup	MA-171193	NM 9524	National Museum Copenhagen	NBA IB	Vandkilde 1996, Aner and Kersten, 1973
Denmark	**#107**	shafthole axe, type Fårdrup	MA-171194	NM 6538	National Museum Copenhagen	NBA IB	Vandkilde 1996, Aner and Kersten, 1973
Denmark	**#108**	shafthole axe, type Fårdrup (d)	MA-171195	NM 6115	National Museum Copenhagen	NBA IB	Vandkilde 1996, Aner and Kersten, 1973
Denmark	**#124**	shafthole axe, type Fårdrup	MA-171340	NM 11201	National Museum Copenhagen	NBA IB	Vandkilde 1996, Aner and Kersten, 1973
Denmark	**#131**	flanged axe, type Oldendorf	MA-171347	B3971	National Museum Copenhagen	NBA IB	Vandkilde 1996, Aner and Kersten, 1973
Denmark	**#153**	shafthole axe, type Fårdrup	MA-171369	St173	Fyns Stiftmuseum	NBA IB	Vandkilde 1996
Denmark	**#185**	flanged axe, type Oldendorf	MA-171401	2089	Moesgaard Museum (Horsens)	NBA IB	Vandkilde 1996
Denmark	**#193**	shafthole axe, type Fårdrup	MA-171409	VHM 13780	Vendsyssel Historiske Museum	NBA IB	Aner and Kersten, 1973
Denmark	**#206**	flanged axe, type Oldendorf	MA-171422	FHM 6504	Forhistorisk Museum Moesgaard	NBA IB	Vandkilde 1996
Denmark	**#208**	flanged axe, type Oldendorf	MA-171424	FHM 6454	Forhistorisk Museum Moesgaard	NBA IB	Vandkilde 1996
Denmark	**#215**	flanged axe, type Oldendorf	MA-171431	FHM 6046	Forhistorisk Museum Moesgaard	NBA IB	Vandkilde 1996
Denmark	**#217**	flanged axe	MA-171433	FHM 6458	Forhistorisk Museum Moesgaard	NBA IB	Vandkilde 1996
Denmark	**#218**	flanged axe	MA-171434	FHM 6421	Forhistorisk Museum Moesgaard	NBA IB	Vandkilde 1996
Denmark	**#220**	flanged axe, type Oldendorf	MA-171436	KHM	Kulturhistorisk Museum Randers	NBA IB	Vandkilde 1996
Denmark	**#221**	flanged axe, type Oldendorf	MA-171437	KHM	Kulturhistorisk Museum Randers	NBA IB	Vandkilde 1996
Denmark	**#251**	shafthole axe Valsømagle	MA-173733	NM B11336	National Museum Copenhagen	NBA IB	unpublished
Denmark	**#303**	flat axe type Bygholm	MA-181031	B13533	National Museum Copenhagen	TRB	Klassen 2000
Denmark	**#304**	flat axe type Bygholm	MA-181032	B13534	National Museum Copenhagen	TRB	Klassen 2000
Dragstrup, Holbo, Fredseriksborg	**#98**	shafthole axe, type Fårdrup (d)	MA-171185	NM 6606	National Museum Copenhagen	NBA IB	Vandkilde 1996, Aner and Kersten, 1973
Dyrehave, Rise, Åbenrå	**#111**	flanged axe, type Underåre	MA-171198	B6980	National Museum Copenhagen	NBA IB	Vandkilde 1996; Aner and Kersten, 1981
Egebakssande, Hundborg, Thisted	**#48**	flanged axe, type Underåre	MA-171135	B1766	National Museum Copenhagen	NBA IB	Vandkilde 1996; Aner and Kersten, 2001
Egelund, Sønderlyng, Viborg	**#202**	flanged axe, type Underaare	MA-171418	B7555	National Museum Copenhagen	NBA IB	Aner and Kersten, 2008; Vandkilde 1996
Egelund, Sønderlyng, Viborg	**#203**	shafthole axe, type Fårdrup	MA-171419	B7554	National Museum Copenhagen	NBA IB	Aner and Kersten, 2008
Egense, Lunde, Odense	**#142**	flanged axe, type Underaare	MA-171358	FSM 3506	Fyns Stiftmuseum	NBA IB	Vandkilde 1996; Aner and Kersten, 1977
Ellehøjgård, Sallinge, Svendborg	**#40**	halbert, type 2	MA-171072	NM 18577	National Museum Copenhagen	LN II	Aner and Kersten, 1977
Elvedgård, Skovby, Odense	**#16**	flanged axe	MA-166667	NM 11919	National Museum Copenhagen	NBA IB	Vandkilde, 1996; Aner and Kersten, 1977
Engemarken, Roskilde, København	**#268**	shafthole axe Valsømagle type	MA-180940	B16780	National Museum Copenhagen	NBA IB	Aner and Kersten, 1973
Ersted By, Hvornum, Ålborg	**#201**	flanged axe, type MaegerkingenV	MA-171417	1081A	Naturhistorisk Museum Aalborg	NBA IB	Vandkilde 1996;
Falster, Denmark	**#63**	flanged axe, type Oldendorf	MA-171150	NM 18590	National Museum Copenhagen	NBA IB	Vandkilde 1996; Aner and Kersten, 1977
Ferritslev, Åsum, Odense	**#44**	flat axe, type 2	MA-171097	A179	Fyns Stiftmuseum	LN I	Aner and Kersten, 1977
Flensted Mark, Gjern, Skanderborg	**#205**	flanged axe, type Underaare	MA-171421	FHM 1005	Forhistorisk Museum Moesgaard	NBA IB	Aner and Kersten, 2014; Vandkilde 1996
Flenstofte, Baag, Odense	**#281**	flanged axe, Anglo-Irish type	MA-180953	NM 26063	National Museum Copenhagen	LN II	Vandkilde 1996; Harbison 1968
Fovslet, Tyrstrup, Vejle	**#180**	flanged axe, type Oldendorf	MA-171396	O-986	Museet på Koldinghus	NBA IB	Vandkilde 1996; Aner and Kersten, 1990
Frankerup, Eggeslevmagle, Sorø	**#257**	belt plate	MA-180919	NM CMXII	National Museum Copenhagen	NBA II	Nørgaard 2018; Aner and Kersten, 1976
Frankerup, Eggeslevmagle, Sorø	**#258**	belt plate	MA-180920	NM CMXII	National Museum Copenhagen	NBA II	Nørgaard 2018; Aner and Kersten, 1976
Frankerup, Eggeslevmagle, Sorø	**#231**	belt plate	MA-173713	NM CMXIII	National Museum Copenhagen	NBA II	Nørgaard 2018; Aner and Kersten, 1976
Freddinge, Beddinge, Scania	**#309**	flanged axe, Anglo-Irish type	MA-181035	ML6573	Lund University Historical Museum	LN II	Vandkilde 1996; Forssander 1936
Fredensborg (near), Lynge, Frederiksborg	**#61**	flanged axe, type Oldendorf	MA-171148	NM 19434	National Museum Copenhagen	NBA IB	Vandkilde 1996, Aner and Kersten, 1973

S1 Table: Complete repository information of the artefacts presented within this study [4, 47, 50, 52, 118, 150-171].

findplace	ID	object/ artefact	lab-no.	collection-no.	museum/ collection	date/ period	context references
Frederiksgave, Sonderby, Baag	**#301**	flanged axe type C3	MA-181029	NM 26071	National Museum Copenhagen	NBA IB	Vandkilde 1996
Frøjk, Hjerm, Ringkøbing	**#269**	shafthole axe Valsømagle type	MA-180941	B11263	National Museum Copenhagen	NBA IB	Aner and Kersten, 1995
Funder, Hids, Viborg	**#97**	flanged axe, type Oldendorf	MA-171184	NM 9572	National Museum Copenhagen	NBA IB	Aner and Kersten, 2008; Vandkilde 1996
Fyn	**#299**	flat axe type Bygholm	MA-181027	B8858	National Museum Copenhagen	TRB	Klassen 2000
Fyrskov, Gram, Haderslev	**#168**	shafthole axe, type Fårdrup	MA-171384	HAM 2601	Museum Sønderjylland Haderslev	NBA IB	Aner and Kersten, 1984
Følle, Ø-Lisbjerg, Randers	**#210**	flanged axe, type Oldendorf	MA-171426	FHM 5102	Forhistorisk Museum Moesgaard	NBA IB	Vandkilde 1996
Førslev (near), Ø Flakkebjerg, Sorø	**#50**	flanged axe, type Underåre	MA-171137	NM 26074	National Museum Copenhagen	NBA IB	Vandkilde 1996; Aner and Kersten, 1976
Føvling, Malt, Ribe	**#11**	flanged axe, type Underåre	MA-166662	NM B3364	National Museum Copenhagen	NBA IB	Vandkilde, 1996; Aner and Kersten, 1986
Gallemose, Støvring, Randers	**#46**	flanged axe, Anglo-Irish type	MA-171133	B3888a	National Museum Copenhagen	LN II	Vandkilde 1996; Harbison 1968
Geishede, Ålborg	**#184**	shafthole axe, type Fårdrup	MA-171400	A252	Horsens Museum	NBA IB	unpublished
Gerdrup, Kirkerup, København	**#248**	belt disc	MA-173730	NM B11459	National Museum Copenhagen	NBA II	Nørgaard 2018
Gerdrup, Kirkerup, København	**#249**	belt disc	MA-173731	NM B11459_2	National Museum Copenhagen	NBA II	Nørgaard 2018
Gern, Gern, Skanderborg	**#214**	flanged axe, type Oldendorf	MA-171430	FHM 2163	Forhistorisk Museum Moesgaard	NBA IB	Aner and Kersten, 2014; Vandkilde 1996
Gislinge, Tuse, Holbæk	**#27**	shafthole axe, type Fårdrup (ud)	MA-166678	NM B4961	National Museum Copenhagen	NBA IB	Aner and Kersten, 1976
Gjedsted, Viborg	**#276**	belt plate	MA-180948	B13260	National Museum Copenhagen	NBA II	Nørgaard 2018; Aner and Kersten, 2008
Gjedsted, Viborg	**#277**	Tutulus	MA-180949	B13261a	National Museum Copenhagen	NBA II	Nørgaard 2018; Aner and Kersten, 2008
Gjedsted, Viborg	**#278**	Tutulus	MA-180950	B13261b	National Museum Copenhagen	NBA II	Nørgaard 2018; Aner and Kersten, 2008
Glæsborg, Nørre, Randers	**#263**	Tutulus	MA-180935	B9535a	National Museum Copenhagen	NBA II	Nørgaard 2018
Glæsborg, Nørre, Randers	**#264**	Tutulus	MA-180936	B9535b	National Museum Copenhagen	NBA II	Nørgaard 2018
Glæsborg, Nørre, Randers	**#265**	belt plate	MA-180937	B9534	National Museum Copenhagen	NBA II	Nørgaard 2018
Grenå (near), Djurs Nørre, Randers	**#219**	flanged axe	MA-171435	VSM 8538	Viborg Stiftsmuseum	NBA IB	Vandkilde 1996
Grubbegård, Bornholm	**#74**	flanged axe, type ext. Oldendorf	MA-171161	NM 22332	National Museum Copenhagen	NBA II	Vandkilde 1996; Aner and Kersten, 1977
Haderslev municipality, Denmark	**#164**	flanged axe, type Oldendorf	MA-171380	HAM 631	Museum Sønderjylland Haderslev	NBA IB	Vandkilde 1996; Aner and Kersten, 1984
Hallenslev, Løve, Holbæk	**#78**	flanged axe, type Oldendorf	MA-171165	B3560	National Museum Copenhagen	NBA IB	Vandkilde 1996; Aner and Kersten, 1976
Havreholm, Lynge, Frederiksborg	**#72**	flanged axe, type Oldendorf	MA-171159	NM 16328	National Museum Copenhagen	NBA IB	Vandkilde 1996, Aner and Kersten, 1973
Hejrede, Musse, Maribo	**#23**	shafthole axe, type Fårdrup (ud)	MA-166674	NM B7248	National Museum Copenhagen	NBA IB	Aner and Kersten, 1977
Hesselhovedgaard, Gislum, Ålborg	**#199**	flanged axe, type Underaare	MA-171415	AR 155	Vesthimmerlands Museum	NBA IB	Vandkilde 1996;
Himlingøje, Bjæverskov, Præstø	**#112**	flanged axe, type Oldendorf	MA-171199	B12537	National Museum Copenhagen	NBA IB	Vandkilde 1996; Aner and Kersten, 1976
Himmestrup, Middelsom, Viborg	**#177**	flanged axe, type Oldendorf	MA-171393	KHM 5484	Kulturhistorisk Museum Randers	NBA IB	Aner and Kersten, 2008; Vandkilde 1996
Hjadstrup, Lunde, Odense	**#34**	flanged axe, type Gallemose	MA-170351	FSM 10183	Fyns Stiftmuseum	LN II	Vandkilde 1996; Aner and Kersten, 1977
Hjerpsted, Højer, Tønder	**#254**	metal-hilted sword, hilt	MA-173736	NM B14457	National Museum Copenhagen	NBA II	Bunnefeld 2016; Aner and Kersten, 1981
Hjerpsted, Højer, Tønder	**#255**	metal-hilted sword, blade	MA-173737	NM B14457	National Museum Copenhagen	NBA II	Bunnefeld 2016; Aner and Kersten, 1981
Hjortslund, Ribe, Ribe	**#176**	flanged axe, type Oldendorf	MA-171392	RIB 5104	Sydvestjyske Museer	NBA IB	Vandkilde 1996;Aner and Kersten, 1986
Hobro (near), Onsild, Randers	**#123**	flanged axe	MA-171339	B2938	National Museum Copenhagen	NBA IB	Vandkilde 1996
Hollufgaard, Åsum, Odense	**#146**	flanged axe, type Oldendorf	MA-171362	FSM 7988	Fyns Stiftmuseum	NBA IB	Vandkilde 1996; Aner and Kersten, 1977
Holmdrup, Sunds, København	**#222**	metal-hilted sword	MA-173703	NM 9441	National Museum Copenhagen	NBA II	Bunnefeld, 2016
Horreby, Falsters Nørre, Maribo	**#137**	flanged axe, type Oldendorf	MA-171353	858	Stiftsmuseum Maribo	NBA IB	Aner and Kersten, 1977
Horsens (near), Nim, Skanderborg	**#211**	flanged axe, type Oldendorf	MA-171427	FHM 845	Forhistorisk Museum Moesgaard	NBA IB	Aner and Kersten, 2014; Vandkilde 1996
Horsens, Nim, Skanderborg	**#93**	flanged axe, type Oldendorf	MA-171180	B8696	National Museum Copenhagen	NBA IB	Aner and Kersten, 2014; Vandkilde 1996
Hundslund, Hads, Aarhus	**#183**	flanged axe, type Oldendorf	MA-171399	A3	Horsens Museum	NBA IB	Aner and Kersten, 2014; Vandkilde 1996
Husum, Sokkelung, København	**#67**	flanged axe, type ext. Oldendorf	MA-171154	B13481	National Museum Copenhagen	NBA II	Vandkilde 1996, Aner and Kersten, 1973
Hvidsted Mose, Børglum, Hjørring	**#191**	flanged axe, type MaegerkingenV	MA-171407	VHM 22062	Vendsyssel Historiske Museum	NBA IB	Vandkilde 1996

S1 Table: Complete repository information of the artefacts presented within this study [4, 47, 50, 52, 118, 150-171].

findplace	ID	object/ artefact	lab-no.	collection-no.	museum/ collection	date/ period	context references
Hvorup Kjær, Kær, Ålborg	**#194**	shafthole axe, type Fårdrup	MA-171410	VHM 12384	Vendsyssel Historiske Museum	NBA IB	Broholm 1952
Høgelund, Gram, Haderslev	**#169**	flanged axe, type Underaare	MA-171385	HAM 1331	Museum Sønderjylland Haderslev	NBA IB	Aner and Kersten, 1984
Højby, Ods, Holbæk	**#9**	flanged axe, type Underåre	MA-166660	NM 5919	National Museum Copenhagen	NBA IB	Vandkilde, 1996; Aner and Kersten, 1976
Højme, Odense, Odense	**#162**	shafthole axe, type Fårdrup	MA-171378	FSM 4665	Fyns Stiftmuseum	NBA IB	Vandkilde 1996; Aner and Kersten, 1977
Højrup, Sallinge, Svendborg	**#144**	flanged axe, type Underaare	MA-171360	FSM 4557	Fyns Stiftmuseum	NBA IB	Vandkilde 1996; Aner and Kersten, 1977
Højsted, Rumperup, Skippinge, Holbæk	**#114**	shafthole axe, type Fårdrup	MA-171330	NM B3092	National Museum Copenhagen	NBA IB	Vandkilde 1996; Aner and Kersten, 1976
Højsted, Rumperup, Skippinge, Holbæk	**#115**	shafthole axe, type Fårdrup	MA-171331	NM B3093	National Museum Copenhagen	NBA IB	Vandkilde 1996; Aner and Kersten, 1976
Højsted, Rumperup, Skippinge, Holbæk	**#116**	shafthole axe, type Fårdrup	MA-171332	NM B3094	National Museum Copenhagen	NBA IB	Vandkilde 1996; Aner and Kersten, 1976
Højsted, Rumperup, Skippinge, Holbæk	**#117**	shafthole axe, type Fårdrup	MA-171333	NM B3095	National Museum Copenhagen	NBA IB	Vandkilde 1996; Aner and Kersten, 1976
Højsted, Rumperup, Skippinge, Holbæk	**#118**	shafthole axe, type Fårdrup	MA-171334	NM B3096a	National Museum Copenhagen	NBA IB	Vandkilde 1996; Aner and Kersten, 1976
Højsted, Rumperup, Skippinge, Holbæk	**#119**	shafthole axe, type Fårdrup	MA-171335	NM B3096b	National Museum Copenhagen	NBA IB	Vandkilde 1996; Aner and Kersten, 1976
Højsted, Rumperup, Skippinge, Holbæk	**#120**	shafthole axe, type Fårdrup	MA-171336	NM B3096c	National Museum Copenhagen	NBA IB	Vandkilde 1996; Aner and Kersten, 1976
Højsted, Rumperup, Skippinge, Holbæk	**#121**	shafthole axe, type Fårdrup	MA-171337	NM B3096d	National Museum Copenhagen	NBA IB	Vandkilde 1996; Aner and Kersten, 1976
Højsted, Rumperup, Skippinge, Holbæk	**#122**	shafthole axe, type Fårdrup	MA-171338	NM B3096e	National Museum Copenhagen	NBA IB	Vandkilde 1996; Aner and Kersten, 1976
Hønsinge, Ods, Holbæk	**#244**	metal-hilted sword	MA-173726	NM B9005	National Museum Copenhagen	NBA II	Bunnefeld 2016; Aner and Kersten, 1976
Hørby (near), Dronninglund, Hjørring	**#190**	flanged axe, type MaegerkingenV	MA-171406	VHM 3497	Vendsyssel Historiske Museum	NBA IB	Vandkilde 1996
Jelling (near of), Tørrild, Vejle	**#95**	flanged axe, type Oldendorf	MA-171182	NM 26107	National Museum Copenhagen	NBA II	Vandkilde 1996; Aner and Kersten, 1990
Juellinge, Lollands Nørre, Maribo	**#26**	shafthole axe, type Fårdrup (d)	MA-166677	NM B10106	National Museum Copenhagen	NBA IB	Aner and Kersten, 1977
Jutland (south-north Jutland)	**#29**	shafthole axe, type Fårdrup (d)	MA-166680	NM B1383	National Museum Copenhagen	NBA IB	unpublished
Jægersborg Hegn, Rundforbi, København	**#238**	neck collar	MA-173720	NM B3059	National Museum Copenhagen	NBA II	Nørgaard 2018
Jægersborg Hegn, Rundforbi, København	**#239**	belt plate	MA-173721	NM B3060	National Museum Copenhagen	NBA II	Nørgaard 2018
Jægerspris, Horns, Frederiksborg	**#77**	flanged axe, type Oldendorf	MA-171164	B13532	National Museum Copenhagen	NBA IB	Vandkilde 1996, Aner and Kersten, 1973
Karup Parish, Lysgaard, Viborg	**#1**	flanged axe, type Aebelnaes	MA-166618	FHM 5014	Forhistorisk Museum Moesgaard	LN II	Aner and Kersten, 2008; Vandkilde, 1996
Ketting, Als Sønder, Sønderborg	**#173**	flanged axe	MA-171389	SØM 360	Odense Bys Museernes Samlinger	NBA IB	Vandkilde 1996;Aner and Kersten, 1981
Kjelstrup, Vilstrup, Haderslev	**#302**	flat axe type Bygholm	MA-181030	B2043	National Museum Copenhagen	TRB	Klassen 2000; Aner and Kersten, 1984
Kolsnap, Gram, Haderslev	**#171**	flanged axe, type Oldendorf	MA-171387	HAM 794	Museum Sønderjylland Haderslev	NBA IB	Aner and Kersten, 1984
Korsør (near), Slagelse, Sorø	**#161**	shafthole axe, type Fårdrup	MA-171377	FSM 5605	Fyns Stiftmuseum	NBA IB	Vandkilde 1996; Aner and Kersten, 1976
Kragenæs, Lollands Nørre, Maribo	**#22**	shafthole axe, type Fårdrup (ud)	MA-166673	NM B5564	National Museum Copenhagen	NBA IB	Aner and Kersten, 1977
Kragsbjerggaard, Odense	**#143**	flanged axe, type Underaare	MA-171359	FSM 2565	Fyns Stiftmuseum	NBA IB	Vandkilde 1996; Aner and Kersten, 1977
Kundby, Tuse, Holbæk	**#14**	flanged axe, type Hüsby	MA-166665	NM 10027	National Museum Copenhagen	NBA IB	Vandkilde, 1996; Aner and Kersten, 1976
Kværndrup, Sunds, Svendborg	**#19**	shafthole axe, type Fårdrup (ud)	MA-166670	NM B11535	National Museum Copenhagen	NBA IB	Aner and Kersten, 1977
Køge (near of), Ramsø, København	**#32**	shafthole axe, type Fårdrup (ud)	MA-166683	NM 11237	National Museum Copenhagen	NBA IB	Aner and Kersten, 1973
Køge (near), Ramsø, København	**#133**	flanged axe, type Underaare	MA-171349	17(1253)	Køge Museum	NBA IB	Vandkilde 1996
Lammefjord, Vallekilde, Ods	**#300**	flanged axe, type C1	MA-181028	B12475	National Museum Copenhagen	NBA IB	Vandkilde 1996
Langeskov, Vindinge, Svendborg	**#152**	flanged axe, type Oldendorf	MA-171368	FSM B5	Fyns Stiftmuseum	NBA IB	Vandkilde 1996; Aner and Kersten, 1977
Langstrup, Asminderød, Frederiksborg	**#266**	belt plate	MA-180938	B2307	National Museum Copenhagen	NBA II	Nørgaard 2018
Langstrup, Asminderød, Frederiksborg	**#267**	belt plate	MA-180939	B2307	National Museum Copenhagen	NBA II	Nørgaard 2018
Lavesgård, Haderslev, Haderslev	**#167**	shafthole axe, type Fårdrup	MA-171383	HAM 4268	Museum Sønderjylland Haderslev	NBA IB	Aner and Kersten, 1984
Ledøje, Smørum, København	**#13**	flanged axe, type Underåre	MA-166664	NM B4208	National Museum Copenhagen	NBA IB	Vandkilde, 1996
Lejre, Volborg, København	**#209**	flanged axe, type Oldendorf	MA-171425	FHM 113	Forhistorisk Museum Moesgaard	NBA IB	Vandkilde 1996
Liengaard, Års, Ålborg	**#200**	shafthole axe, type Fårdrup	MA-171416	AR 100	Vesthimmerlands Museum	NBA IB	Vandkilde 1996; Forssander 1936
Lille Halkjær, Hind, Ringkøbing	**#91**	flanged axe, type Oldendorf	MA-171178	B7377	National Museum Copenhagen	NBA IB	Vandkilde 1996; Aner and Kersten, 1995

S1 Table: Complete repository information of the artefacts presented within this study [4, 47, 50, 52, 118, 150-171].

findplace	ID	object/ artefact	lab-no.	collection-no.	museum/ collection	date/ period	context references
Lille-Dalbygård, Hatting, Vejle	**#15**	flanged axe, type Underåre	MA-166666	NM B12048	National Museum Copenhagen	NBA IB	Vandkilde, 1996; Aner and Kersten, 1990
Lumby, Lunde, Odense	**#140**	flanged axe, type Oldendorf	MA-171356	FSM 7100	Fyns Stiftmuseum	NBA IB	Vandkilde 1996; Aner and Kersten, 1977
Lund (near), Sweden	**#307**	spoon-shaped axe, type B1	MA-181033	ML2822	Lund University Historical Museum	NBA IB	Vandkilde 1996; Oldeberg 1974
Lund (near), Sweden	**#308**	spoon-shaped axe, type B2	MA-181034	ML2823	Lund University Historical Museum	NBA IB	Vandkilde 1996; Oldeberg 1974
Lund, Stevs, Præstø	**#73**	flanged axe, type Oldendorf	MA-171160	B5919	National Museum Copenhagen	NBA IB	Vandkilde 1996; Aner and Kersten, 1976
Lunding, Haderslev, Haderslev	**#170**	flanged axe, type Oldendorf	MA-171386	HAM 4454	Museum Sønderjylland Haderslev	NBA IB	Aner and Kersten, 1984
Lyngby, Hellum, Ålborg	**#198**	shafthole axe, type Fårdrup	MA-171414	ÅHM 3518	Forhistorisk Museum Moesgaard	NBA IB	Vandkilde 1996; Forssander 1936
Lyngå, Sabro, Aarhus	**#7**	flanged axe	MA-166658	NM 18186	National Museum Copenhagen	NBA IB	Aner and Kersten, 2014; Vandkilde, 1996
Lysabildgaard, Als Sømnder, Sønderborg	**#156**	flanged axe, type Underaare	MA-171372	CM69	Fyns Stiftmuseum	NBA IB	Vandkilde 1996; Aner and Kersten, 1981
Löddeköpinge, Harjagers, Scania	**#311**	flanged axe, Anglo-Irish type	MA-181037	ML11056	Lund University Historical Museum	LN II	Vandkilde 1996; Forssander 1936
Mellem Mølgård, Vennebjerg, Hjørring	**#192**	shafthole axe, type Fårdrup	MA-171408	VHM 22103	Vendsyssel Historiske Museum	NBA IB	Broholm 1952
Mosgaard, Taarup, Fjends	**#3**	flat axe, type 4	MA-166624	NM B5155	National Museum Copenhagen	TRB	Vandkilde, 1996
Moskjaer, Sønderhald, Randers	**#5**	flanged axe, type Underåre	MA-166656	NM B4077	National Museum Copenhagen	NBA IB	Vandkilde, 1996
Nahe Ellinge, Vindinge, Svendborg	**#81**	flanged axe, type Oldendorf	MA-171168	B3108	National Museum Copenhagen	NBA IB	Vandkilde 1996; Aner and Kersten, 1977
Nahe Hvedholm, Sallinge, Svendborg	**#88**	flanged axe, type Oldendorf	MA-171175	B1461	National Museum Copenhagen	NBA IB	Vandkilde 1996; Aner and Kersten, 1977
Neble, Slagelse, Sorø	**#21**	shafthole axe, type Fårdrup (ud)	MA-166672	NM B10894	National Museum Copenhagen	NBA IB	Aner and Kersten, 1976
Nistrup, Horns, Hjørring	**#6**	flanged axe, type Underåre	MA-166657	NM B965	National Museum Copenhagen	NBA IB	Vandkilde, 1996
Nord Fünen, Odense	**#149**	flanged axe, type Underaare	MA-171365	CM100	Fyns Stiftmuseum	NBA IB	Vandkilde 1996; Aner and Kersten, 1977
Northern Fyn, Odense	**#154**	flanged axe, type Oldendorf	MA-171370	CM68	Fyns Stiftmuseum	NBA IB	Vandkilde 1996; Aner and Kersten, 1977
Norup, Lunde, Odense	**#139**	flanged axe, type Underaare	MA-171355	FSM CM141	Fyns Stiftmuseum	NBA IB	Vandkilde 1996; Aner and Kersten, 1977
Nysted, Musse, Maribo	**#135**	flanged axe, type Oldendorf	MA-171351	NM 547	National Museum Copenhagen	NBA IB	Vandkilde 1996; Aner and Kersten, 1977
Odense, Odense	**#150**	flanged axe, type Underaare	MA-171366	FSM 5268	Fyns Stiftmuseum	NBA IB	Vandkilde 1996; Aner and Kersten, 1977
Odsherred, Ods, Holbæk	**#49**	flanged axe, type Underåre	MA-171136	B10792	National Museum Copenhagen	NBA IB	Vandkilde 1996; Aner and Kersten, 1976
Oppe-Sundby, Lynge, Frederiksborg	**#109**	flanged axe, type Underåre	MA-171196	B11037	National Museum Copenhagen	NBA IB	Vandkilde 1996, Aner and Kersten, 1973
Osted, Voldborg, København	**#38**	flat axe, type 1	MA-171062	NM 13810	National Museum Copenhagen	TRB	Vandkilde 1996, Aner and Kersten, 1973
Over Vindinge, Hammer, Præsto	**#306**	spearhead	MA-192837	NM B26886	National Museum Copenhagen	NBA IB	unpublished
Over-Blåkrog, Lundtoft, Åbenrå	**#174**	flanged axe, type Underaare	MA-171390	AA 962	Museum Sønderjylland	NBA IB	Vandkilde 1996;Aner and Kersten, 1981
Over-Fidde, Vester-Holm, Ribe	**#89**	flanged axe, type Oldendorf	MA-171176	B1979	National Museum Copenhagen	NBA IB	Vandkilde 1996; Aner and Kersten, 1986
Over-Jerstal, Gram, Haderslev	**#165**	flanged axe, type Oldendorf	MA-171381	HAM 3824	Museum Sønderjylland Haderslev	NBA IB	Vandkilde 1996; Aner and Kersten, 1984
Pandrup (near), Refs, Thisted	**#8**	flanged axe, type Underåre	MA-166659	NM B6702	National Museum Copenhagen	NBA IB	Vandkilde, 1996; Aner and Kersten, 200[illegible]
Pillemark, Samsø, Holbæk	**#18**	shafthole axe, type Fårdrup (ud)	MA-166669	NM B12015	National Museum Copenhagen	NBA IB	Aner and Kersten, 1976
Ramsø, Tune, København	**#57**	flanged axe, type Oldendorf	MA-171144	NM 7782	National Museum Copenhagen	NBA IB	Vandkilde 1996, Aner and Kersten, 1973
Ravnsby, Lollands Nørre, Maribo	**#62**	flanged axe, type Oldendorf	MA-171149	B5501	National Museum Copenhagen	NBA IB	Vandkilde 1996; Aner and Kersten, 1977
Refs-Vindinge, Vindinge, Svendborg	**#151**	flanged axe, type Underaare	MA-171367	20.4.1885	Fyns Stiftmuseum	NBA IB	Vandkilde 1996; Aner and Kersten, 1977
Ringgive, Nørvang	**#298**	shafthole axe Fårdrup type	MA-181026	NM 2868	National Museum Copenhagen	NBA IB	Vandkilde 1996; Forssander 1936
Risgårde, Lysgård, Viborg	**#96**	flanged axe, type Oldendorf	MA-171183	NM 8132	National Museum Copenhagen	NBA IB	Aner and Kersten, 2008; Vandkilde 1996
Rye, Gorlev, Holbaek	**#256**	belt plate	MA-180918	B7615	National Museum Copenhagen	NBA II	Nørgaard 2018
Raagelund, Åsum, Odense	**#148**	flanged axe, type Oldendorf	MA-171364	CM129	Fyns Stiftmuseum	NBA IB	Vandkilde 1996; Aner and Kersten, 1977
Sadderup, Skads, Ribe	**#178**	flanged axe	MA-171394	ESB 178	Sydvestjyske Museer	NBA IB	Vandkilde 1996; Aner and Kersten, 1986
Samsø, Samsø, Holbæk	**#155**	flanged axe, type Oldendorf	MA-171371	5.11.1886	Fyns Stiftmuseum	NBA IB	Vandkilde 1996; Aner and Kersten, 1976
Sandby, Lollands Nørre, Maribo	**#130**	flanged axe, type Oldendorf	MA-171346	NM 3105	National Museum Copenhagen	NBA IB	Vandkilde 1996; Aner and Kersten, 1977
Sanderum, Odense, Odense	**#163**	shafthole axe, type Fårdrup	MA-171379	FSM 2199	Fyns Stiftmuseum	NBA IB	Vandkilde 1996; Aner and Kersten, 1977

S1 Table: Complete repository information of the artefacts presented within this study [4, 47, 50, 52, 118, 150-171].

findplace	ID	object/ artefact	lab-no.	collection-no.	museum/ collection	date/ period	context references
Selchausdal, Løve, Holbæk	**#279**	flanged axe, Anglo-Irish type	MA-180951	B5310	National Museum Copenhagen	LN II	Vandkilde 1996; Harbison 1968
Selchausdal, Løve, Holbæk	**#280**	flanged axe, Anglo-Irish type	MA-180952	NM 4558	National Museum Copenhagen	LN II	Vandkilde 1996; Harbison 1968
Signalbakkemarken Fleskum, Ålborg	**#197**	flanged axe, type Underaare	MA-171413	ÅHM 3563	Forhistorisk Museum Moesgaard	NBA IB	Vandkilde 1996;
Silkeborg (near), Gjern, Skanderborg	**#213**	flanged axe, type Oldendorf	MA-171429	FHM 5154	Forhistorisk Museum Moesgaard	NBA IB	Aner and Kersten, 2014; Vandkilde 1996
Silkeborg, Gjern, Skanderborg	**#166**	flanged axe, type Underaare	MA-171382	HAD 8768	Museum Sønderjylland Haderslev	NBA IB	Aner and Kersten, 2014; Vandkilde 1996
Skals, Rinds, Viborg	**#90**	flanged axe, type Oldendorf	MA-171177	B8703	National Museum Copenhagen	NBA IB	Aner and Kersten, 2008; Vandkilde 1996
Skrydstrup, Gram, Haderslev	**#252**	metal-hilted sword	MA-173734	NM B13743	National Museum Copenhagen	NBA II	Bunnefeld 2016; Aner and Kersten, 1984
Skrydstrup, Gram, Haderslev	**#253**	metal-hilted sword	MA-173735	NM B13743	National Museum Copenhagen	NBA II	Bunnefeld 2016; Aner and Kersten, 1984
Skærbæk, Elbo, Vejle	**#179**	flanged axe, type MaegerkingenV	MA-171395	O-3234	Museet på Koldinghus	NBA IB	Vandkilde 1996; Aner and Kersten, 1990
Skørring, Framlev, Aarhus	**#216**	flanged axe, type Underaare	MA-171432	FHM 5846	Forhistorisk Museum Moesgaard	NBA IB	Aner and Kersten, 2014; Vandkilde 1996
Slusegård, Sønder, Bornholm	**#35**	flat axe, type 1	MA-171059	B3459	National Museum Copenhagen	TRB	Vandkilde 1996; Aner and Kersten, 1977
Smørumovre, Smørum, København	**#245**	metal-hilted sword, hilt	MA-173727	NM B2110	National Museum Copenhagen	NBA II	Bunnefeld 2016
Smørumovre, Smørum, København	**#246**	metal-hilted sword, blade	MA-173728	NM B2110	National Museum Copenhagen	NBA II	Bunnefeld 2016
Smørumovre, Smørum, København	**#247**	metal-hilted sword	MA-173729	NM B2110	National Museum Copenhagen	NBA II	Bunnefeld 2016
Snekkerup, V-Flakkebjerg, Sorø	**#60**	flanged axe, type Oldendorf	MA-171147	B13584	National Museum Copenhagen	NBA IB	Vandkilde 1996; Aner and Kersten, 1976
Snoldelev, Tune, København	**#69**	flanged axe, type ext. Oldendorf	MA-171156	NM 12167	National Museum Copenhagen	NBA II	Vandkilde 1996, Aner and Kersten, 1973
Sperrringsjö, Hundborg, Thisted	**#92**	flanged axe, type Oldendorf	MA-171179	B14075	National Museum Copenhagen	NBA IB	Vandkilde 1996; Aner and Kersten, 2001
Stangerup, Falster Nørre, Maribo	**#113**	flanged axe, type Underåre	MA-171200	B1609	National Museum Copenhagen	NBA IB	Vandkilde 1996; Aner and Kersten, 1977
Stavreby, Bårse, Præsto	**#53**	flanged axe, type Underåre	MA-171140	B6978	National Museum Copenhagen	NBA IB	Vandkilde 1996; Aner and Kersten, 1976
Stensbygaard, Baarse, Præstø	**#157**	flanged axe, type Oldendorf	MA-171373	Mi403	Fyns Stiftmuseum	NBA IB	Vandkilde 1996; Aner and Kersten, 1976
Stevning, Als Nørre, Svendborg	**#172**	flanged axe, type Oldendorf	MA-171388	SØM 368	Odense Bys Museernes Samlinger	NBA IB	Vandkilde 1996; Aner and Kersten, 1977
Stevns, Stevns, Præstø	**#59**	flanged axe, type Oldendorf	MA-171146	MMCCII	National Museum Copenhagen	NBA IB	Vandkilde 1996; Aner and Kersten, 1976
Stige (near), Lunde, Odense	**#145**	flanged axe, type Oldendorf	MA-171361	FSM 3280	Fyns Stiftmuseum	NBA IB	Vandkilde 1996; Aner and Kersten, 1977
Stokkebro, Djurs Nørre, Randers	**#94**	flanged axe, type Underåre	MA-171181	B14017	National Museum Copenhagen	NBA IB	Vandkilde 1996
Store Karleby, Volborg, København	**#76**	flanged axe, type ext. Oldendorf	MA-171163	B6692	National Museum Copenhagen	NBA II	Vandkilde 1996
Store Valby, Sømme, Købenahvn	**#68**	flanged axe, type Oldendorf	MA-171155	B638	National Museum Copenhagen	NBA IB	Vandkilde 1996, Aner and Kersten, 1973
Store-Heddinge, Stevens, Præstø	**#282**	flanged axe, Anglo-Irish type	MA-180954	MLXIX	National Museum Copenhagen	LN II	Vandkilde 1996; Harbison 1968
Store-Heddinge, Stevens, Præstø	**#283**	flanged axe, Anglo-Irish type	MA-180955	MLXXa	National Museum Copenhagen	LN II	Vandkilde 1996; Harbison 1968
Store-Heddinge, Stevens, Præstø	**#284**	flanged axe, Anglo-Irish type	MA-180956	MLXXb	National Museum Copenhagen	LN II	Vandkilde 1996; Harbison 1968
Store-Heddinge, Stevens, Præstø	**#285**	flanged axe, Anglo-Irish type	MA-180957	MLXXc	National Museum Copenhagen	LN II	Vandkilde 1996; Harbison 1968
Strøby Egede, Stevns, Præstø	**#71**	flanged axe, type Oldendorf	MA-171158	B10581	National Museum Copenhagen	NBA IB	Vandkilde 1996; Aner and Kersten, 1976
Stude, Slagelse, Sorø	**#51**	flanged axe, type Oldendorf	MA-171138	B12199	National Museum Copenhagen	NBA IB	Vandkilde 1996; Aner and Kersten, 1976
Støvel, Hjerm, Ringkøbing	**#28**	shafthole axe, type Fårdrup (d)	MA-166679	NM B4938	National Museum Copenhagen	NBA IB	Aner and Kersten, 1995
Svallerup, Ars, Holbæk	**#10**	flanged axe	MA-166661	NM B10153	National Museum Copenhagen	NBA IB	Vandkilde, 1996; Aner and Kersten, 1976
Svenstrup, Tårnborg, Sorø	**#224**	belt disc	MA-173705	NM 10935a	National Museum Copenhagen	NBA II	Nørgaard 2018; Aner and Kersten, 1976
Svenstrup, Tårnborg, Sorø	**#225**	belt disc	MA-173706	NM 10935b	National Museum Copenhagen	NBA II	Nørgaard 2018; Aner and Kersten, 1976
Svenstrup, Tårnborg, Sorø	**#226**	belt disc	MA-173707	NM 10935c	National Museum Copenhagen	NBA II	Nørgaard 2018; Aner and Kersten, 1976
Svenstrup, Tårnborg, Sorø	**#227**	belt disc	MA-173708	NM 10938a	National Museum Copenhagen	NBA II	Nørgaard 2018; Aner and Kersten, 1976
Svenstrup, Tårnborg, Sorø	**#228**	belt disc	MA-173709	NM 10938b	National Museum Copenhagen	NBA II	Nørgaard 2018; Aner and Kersten, 1976
Svenstrup, Tårnborg, Sorø	**#229**	belt disc	MA-173710	NM 10938c	National Museum Copenhagen	NBA II	Nørgaard 2018; Aner and Kersten, 1976
Søborg Sø, Holbo, Frederiksborg	**#64**	flanged axe, type ext.Oldendorf	MA-171151	B11381	National Museum Copenhagen	NBA II	Vandkilde 1996, Aner and Kersten, 1973
Søby Hede, Fjends, Viborg	**#42**	flat axe, type 1	MA-171088	B6984	National Museum Copenhagen	TRB	Aner and Kersten, 2008; Vandkilde 1996

S1 Table: Complete repository information of the artefacts presented within this study [4, 47, 50, 52, 118, 150-171].

findplace	ID	object/ artefact	lab-no.	collection-no.	museum/ collection	date/ period	context references
Søby Hede, Fjends, Viborg	**#43**	armspiral	MA-171089	B6985	National Museum Copenhagen	TRB	Aner and Kersten, 2008; Klassen 2000
Søndersø, Skovby, Odense	**#47**	flanged axe, type Underåre	MA-171134	B291	National Museum Copenhagen	NBA IB	Vandkilde 1996; Aner and Kersten, 1977
Tiselholt, Gudme, Svendborg	**#240**	metal-hilted sword, blade	MA-173722	NM B121	National Museum Copenhagen	NBA II	Bunnefeld 2016; Aner and Kersten, 1977
Tiselholt, Gudme, Svendborg	**#241**	metal-hilted sword	MA-173723	NM B121	National Museum Copenhagen	NBA II	Bunnefeld 2016; Aner and Kersten, 1977
Tiselholt, Gudme, Svendborg	**#242**	metal-hilted sword	MA-173724	NM B120	National Museum Copenhagen	NBA II	Bunnefeld 2016; Aner and Kersten, 1977
Tiselholt, Gudme, Svendborg	**#243**	metal-hilted sword	MA-173725	NM B120	National Museum Copenhagen	NBA II	Bunnefeld 2016; Aner and Kersten, 1977
Tobøl, Malt, Ribe	**#223**	metal-hilted sword	MA-173704	NM B5760	National Museum Copenhagen	NBA II	Bunnefeld, 2016; Aner and Kersten, 1986
Tøjstrup, Sønderhalde, Randers	**#45**	flat axe, type 2	MA-171130	KHM 1560	Kulturhistorisk Museum Randers	LN I	Vandkilde 1996
Tågelund, Jerslev, Vejle	**#99**	shafthole axe, type Fårdrup (d)	MA-171186	NM 18154	National Museum Copenhagen	NBA IB	Vandkilde 1996; Aner and Kersten, 1990
Tårnholm, Slagelse, Sorø	**#31**	shafthole axe, type Fårdrup (d)	MA-166682	NM 26013	National Museum Copenhagen	NBA IB	Vandkilde 1996; Aner and Kersten, 1976
Tåsinge, Sunds, Svendborg	**#141**	flanged axe, type Smoerumovre	MA-171357	FSM 5261	Fyns Stiftmuseum	NBA II	Vandkilde 1996; Aner and Kersten, 1977
Udby, Mønbo, Præstø	**#66**	flanged axe, type Oldendorf	MA-171153	B5559	National Museum Copenhagen	NBA IB	Vandkilde 1996; Aner and Kersten, 1976
Uhe, Nørvang, Vejle	**#125**	shafthole axe, type Fårdrup	MA-171341	NM 2868	National Museum Copenhagen	NBA IB	Vandkilde 1996; Aner and Kersten, 1990
Ulfshale, Mønsted, Præstø	**#134**	flanged axe, type Oldendorf	MA-171350	8665	Møns Museum	NBA IB	Vandkilde 1996; Aner and Kersten, 1976
Ullerslev, Vindinge, Svendborg	**#271**	spearhead	MA-180943	NM 8850a	National Museum Copenhagen	NBA II	Aner and Kersten, 1977
Ullerslev, Vindinge, Svendborg	**#272**	spearhead	MA-180944	NM 8850b	National Museum Copenhagen	NBA II	Aner and Kersten, 1977
Underåre, Børglum, Hjørring	**#187**	flanged axe, type Underaare	MA-171403	VHM 22366	Vendsyssel Historiske Museum	NBA IB	Vandkilde 1996
Underåre, Børglum, Hjørring	**#188**	flanged axe, type Underaare	MA-171404	VHM 22365B	Vendsyssel Historiske Museum	NBA IB	Vandkilde 1996
Underåre, Børglum, Hjørring	**#189**	flanged chissel	MA-171405	VHM 22365A	Vendsyssel Historiske Museum	NBA IB	Vandkilde 1996
Valby, Holbo, Frederiksborg	**#79**	flanged axe, type ext. Oldendorf	MA-171166	NM Z689	National Museum Copenhagen	NBA II	Vandkilde 1996, Aner and Kersten, 1973
Valore, Ramsø, København	**#33**	flanged axe, type Værslev	MA-170348	B3370	National Museum Copenhagen	LN II	Vandkilde 1996, Aner and Kersten, 1973
Valsømagle, Ringsted, Sorø	**#259**	shafthole axe Valsømagle type	MA-180931	B3949	National Museum Copenhagen	NBA IB	Aner and Kersten, 1976
Valsømagle, Ringsted, Sorø	**#260**	shafthole axe Valsømagle type	MA-180932	B3473	National Museum Copenhagen	NBA IB	Aner and Kersten, 1976
Valsømagle, Ringsted, Sorø	**#261**	shafthole axe Valsømagle type	MA-180933	B7520	National Museum Copenhagen	NBA IB	Aner and Kersten, 1976
Valsømagle, Ringsted, Sorø	**#262**	spearhead	MA-180934	B3951	National Museum Copenhagen	NBA IB	Aner and Kersten, 1976
Vellinge Mose, Bederslev, Odense	**#232**	belt disc	MA-173714	NM 25789_2	National Museum Copenhagen	NBA II	Nørgaard 2018; Aner and Kersten, 1977
Vellinge Mose, Bederslev, Odense	**#233**	belt disc	MA-173715	NM 25789_1	National Museum Copenhagen	NBA II	Nørgaard 2018; Aner and Kersten, 1978
Vellinge Mose, Bederslev, Odense	**#234**	belt plate	MA-173716	NM 25788	National Museum Copenhagen	NBA II	Nørgaard 2018; Aner and Kersten, 1979
Vellinge Mose, Bederslev, Odense	**#235**	neck collar	MA-173717	NM 25787	National Museum Copenhagen	NBA II	Nørgaard 2018; Aner and Kersten, 1980
Vellinge Mose, Bederslev, Odense	**#236**	belt plate	MA-173718	NM B2654	National Museum Copenhagen	NBA II	Nørgaard 2018; Aner and Kersten, 1981
Vendsyssel, Hjørring	**#204**	flanged axe, type Oldendorf	MA-171420	VHM 1981/14	Vendsyssel Historiske Museum	NBA IB	Vandkilde 1996
Vendsyssel, København, København	**#250**	belt disc	MA-173732	NM B4753_890	National Museum Copenhagen	NBA II	Nørgaard 2018
Vesterelling, Middelsom, Viborg	**#87**	flanged axe, type Underåre	MA-171174	B429	National Museum Copenhagen	NBA IB	Vandkilde 1996
Vestergård, Slagelse, Sorø	**#75**	flanged axe, type MaegerkingenV	MA-171162	B9511	National Museum Copenhagen	NBA IB	Vandkilde 1996; Aner and Kersten, 1976
Vesterå, Børglum, Hjørring	**#270**	shafthole axe	MA-180942	NM 19176	National Museum Copenhagen	NBA II	unpublished
Viborg, Nørlyng, Viborg	**#36**	flat axe, type 3	MA-171060	NM 26057	National Museum Copenhagen	TRB	Aner and Kersten, 2008; Vandkilde 1996
Viborg, Nørlyng, Viborg	**#37**	flat axe, type 3	MA-171061	NM 26058	National Museum Copenhagen	TRB	Aner and Kersten, 2008; Vandkilde 1996
Viby, Bjerge, Odense	**#110**	flanged axe, type Oldendorf	MA-171197	B978	National Museum Copenhagen	NBA IB	Vandkilde 1996; Aner and Kersten, 1977
Virring, Sønderhald, Randers	**#286**	flanged axe, Torsted-Tindsdahl	MA-180962	B3963	National Museum Copenhagen	NBA IA	Vandkilde 1996; Harbison 1969
Virring, Sønderhald, Randers	**#287**	flanged axe, Virring type	MA-180963	B3962	National Museum Copenhagen	NBA IA	Vandkilde 1996
Vognserup Enge, Holbæk	**#291**	belt plate	MA-180967	VM1680KC	National Museum Copenhagen	NBA II	Nørgaard 2018; Frost 2011
Vognserup Enge, Holbæk	**#292**	belt plate	MA-180968	VM1680KD	National Museum Copenhagen	NBA II	Nørgaard 2018; Frost 2011

S1 Table: Complete repository information of the artefacts presented within this study [4, 47, 50, 52, 118, 150-171].

findplace	ID	object/ artefact	lab-no.	collection-no.	museum/ collection	date/ period	context references
Vognserup Enge, Holbæk	**#293**	small belt plate	MA-180969	VM1680AH	National Museum Copenhagen	NBA II	Nørgaard 2018; Frost 2011
Vognserup Enge, Holbæk	**#294**	small belt plate	MA-180970	VM1680AL	National Museum Copenhagen	NBA II	Nørgaard 2018; Frost 2011
Vognserup Enge, Holbæk	**#295**	neck collar	MA-180971	VM1680KF	National Museum Copenhagen	NBA II	Nørgaard 2018; Frost 2011
Vognserup Enge, Holbæk	**#296**	neck collar	MA-180972	VM1680KE	National Museum Copenhagen	NBA II	Nørgaard 2018; Frost 2011
Vognserup Enge, Holbæk	**#297**	belt plate, eyelet	MA-181023	VM 1680 KJ	National Museum Copenhagen	NBA II	Nørgaard 2018; Frost 2011
Vordinborg, Baarse, Præstø	**#132**	flanged axe, type Oldendorf	MA-171348	NM 20983	National Museum Copenhagen	NBA IB	Vandkilde 1996; Aner and Kersten, 1976
Vorup, Vejen, Ribe	**#230**	belt disc	MA-173711	NM B6623_1	National Museum Copenhagen	NBA II	Nørgaard 2018; Aner and Kersten, 1986
Vålse, Falsters Nørre, Maribo	**#24**	shafthole axe, type Fårdrup (d)	MA-166675	NM B8025	National Museum Copenhagen	NBA IB	Aner and Kersten, 1977
Zealand, Danmark	**#195**	flanged axe, type Underaare	MA-171411	VHM 12325	Vendsyssel Historiske Museum	NBA IB	Vandkilde 1996; Aner and Kersten 1976
Æbelnæs, Mønbo, Præstø	**#39**	flanged axe, type Æbelnæs	MA-171063	B494	National Museum Copenhagen	LN II	Vandkilde 1996; Aner and Kersten, 1976
Ølby, Nordhøj, Ramsø, København	**#273**	metal-hilted sword	MA-180945	B2201	National Museum Copenhagen	NBA II	Nørgaard 2018; Reiter et al. 2019
Ølby, Nordhøj, Ramsø, København	**#274**	neck collar	MA-180946	B2200	National Museum Copenhagen	NBA II	Nørgaard 2018; Reiter et al. 2019
Ølby, Nordhøj, Ramsø, København	**#275**	belt plate	MA-180947	B2202	National Museum Copenhagen	NBA II	Nørgaard 2018; Reiter et al. 2019
Ølby, Ramsø, København	**#54**	flanged axe, type Underåre	MA-171141	B6350	National Museum Copenhagen	NBA IB	Vandkilde 1996, Aner and Kersten, 1973
Østofte, Fuglse, Maribo	**#136**	flanged axe, type Oldendorf	MA-171352	546	Stiftsmuseum Maribo	NBA IB	Vandkilde 1996; Aner and Kersten, 1977
Aalsrode Mark, Djurs Sønder, Randers	**#207**	shafthole axe, type Fårdrup	MA-171423	FHM 1884	Forhistorisk Museum Moesgaard	NBA IB	Vandkilde 1996; Forssander 1936
Ås Mark, Tvingstrup, Skanderborg	**#20**	shafthole axe, type Fårdrup (ud)	MA-166671	NM B11335	National Museum Copenhagen	NBA IB	Aner and Kersten, 2014
